# Association between physical activity and incident dementia among individuals with hypertension

**DOI:** 10.3389/fnagi.2026.1833102

**Published:** 2026-09-15

**Authors:** Chenguang Yang, Yejing Zhao, Jingkuo Li, Lubi Lei, Yi Li, Wei Wang, XuYang Meng, ChenXi Xia, Lihua Zhang, Xiang Wang, Fang Wang

**Affiliations:** 1 Department of Cardiology, Beijing Hospital, National Center of Gerontology, Institute of Geriatric Medicine, Chinese Academy of Medical Sciences, Beijing, China; 2 Department of Cardiology, Beijing Anzhen Hospital, Capital Medical University, Beijing, China; 3 National Clinical Research Center for Cardiovascular Diseases, Fuwai Hospital, National Center for Cardiovascular Diseases, Chinese Academy of Medical Sciences and Peking Union Medical College, Beijing, China; 4 Center for Clinical and Epidemiologic Research, Beijing Anzhen Hospital, Capital Medical University, Beijing Institute of Heart, Lung and Blood Vessel Diseases, Beijing, China; 5 Department of Gerontology, Beijing Hospital, National Center of Gerontology, Institute of Geriatric Medicine, Chinese Academy of Medical Sciences, Beijing, China

**Keywords:** accelerometer, dementia, hypertension, physical activity, risk factors

## Abstract

**Objectives:**

Evidence on the association between physical activity (PA) and incident dementia among individuals with hypertension remains limited. This study examined the associations between different PA types and intensities and dementia risk in this population.

**Methods:**

Data from questionnaire-based and accelerometer-based cohorts were analyzed. PA was categorized into quartiles of light-intensity (LPA), moderate-intensity (MPA), vigorous-intensity (VPA), moderate-to-vigorous-intensity PA (MVPA), and total PA (TPA). PA domains included leisure time, housework, transportation, and occupational activities. Incident dementia was identified using hospital inpatient and death records, which may not capture milder dementia cases and classified as early- or late-onset by age at diagnosis. Cox proportional hazards models were used.

**Results:**

In the accelerometer-based cohort of 47,861 hypertensive participants, 574 (1.2%) developed dementia over a median of 8.1 years. Compared with the lowest quartile, the highest quartile of MPA (HR 0.41, 95% CI 0.31–0.54), VPA (HR 0.34, 95% CI 0.25–0.46), MVPA (HR 0.43, 95% CI 0.32–0.57), and TPA (HR 0.63, 95% CI 0.49–0.82) had lower risks of incident dementia. In the questionnaire-based cohort, 5,647 (2.5%) cases occurred over a median of 13.7 years. Higher PA levels, particularly leisure-time and housework-related PA, were consistently tied to a lower risk estimate. MPA showed a more pronounced risk reduction for early-onset than late-onset dementia (ratio of HR 0.69, 95% CI 0.50–0.96).

**Conclusion:**

Higher levels of MPA, MVPA, VPA, and TPA are associated with a lower risk profile for dementia among individuals with hypertension, with variations observed across activity domains and age of onset. However, the reliance on hospital and death records for dementia ascertainment may underestimate the true burden by missing milder cases. These findings demonstrate associations rather than causal effects and require confirmation in intervention studies.

## Introduction

Dementia affected approximately 57 million people worldwide in 2019 and is projected to affect 152 million people by 2050 (Gorelick et al., 2011; Livingston et al., 2017; GBD 2019 Dementia Forecasting Collaborators, 2022). Hypertension has been recognized as the most important risk factor for developing stroke, cognitive impairment, and dementia (Sierra et al., 2011; Meschia et al., 2014; Norton et al., 2014). Since current pharmacological treatments cannot cure or reverse dementia and can only provide limited symptom relief, there is growing emphasis on identifying potential modifiable risk factors that could prevent dementia onset and progression in hypertensive individuals.

Physical activity (PA) can effectively reduce the risk of dementia and has been suggested as a modifiable risk factor to prevent dementia (Hogan et al., 2008; Livingston et al., 2017; Oh and Rabins, 2019). However, research on the associations between PA and the risk of dementia has mostly been conducted in the general population. One meta-analysis showed that, compared with the lowest PA category, higher levels of PA were associated with a lower risk of all-cause dementia and Alzheimer’s disease (Iso-Markku et al., 2022). Another cohort study found that higher levels of accelerometer-measured PA were associated with a lower risk of incident dementia (Petermann-Rocha et al., 2021). Though PA has been recommended in individuals with hypertension to reduce blood pressure (BP) and cardiovascular events (National Institute for Health and Care Excellence (NICE), 2023), whether the inverse association between PA and dementia observed in the general population also applies to individuals with hypertension remains uncertain. This is clinically important because hypertension is an established risk factor for dementia and may contribute to dementia through vascular and cerebrovascular mechanisms, while individuals with hypertension may also differ from the general population in their cardiovascular risk profile, comorbidity burden, and use of antihypertensive medications. These factors may influence the association between PA and dementia and limit the direct generalizability of findings from the general population to individuals with hypertension. Norton et al. (2014) estimated that physical inactivity accounted for approximately 6.5% of the population-attributable risk of late-life Alzheimer’s disease, whereas evidence regarding the role of PA in early-onset dementia remains limited. Therefore, whether higher levels of PA, particularly objectively measured PA, are associated with lower risks of early-onset and late-onset dementia among individuals with hypertension remains insufficiently understood. Addressing this question may provide evidence to inform lifestyle-based dementia prevention in this high-risk population.

This study was a secondary analysis of data from the UK Biobank, a large prospective population-based cohort study. We aimed to examine the association between PA and dementia in two cohorts: a questionnaire-based cohort and an accelerometer-based cohort. Self-reported PA can capture different domains and types of physical activity that may not be fully captured by device-based measurements, but it is potentially subject to recall and reporting bias. In contrast, accelerometer-measured PA provides an objective assessment of activity intensity and duration. Therefore, examining both self-reported and accelerometer-measured PA may provide complementary evidence and help assess the robustness and consistency of the associations between PA and dementia. We also used the questionnaire-based cohort to examine the association between various types of PA and the risk of dementia and to examine differences between early-onset and late-onset dementia. Furthermore, different intensities of PA may confer different physiological stimuli and levels of energy expenditure. Examining moderate and vigorous PA separately, as well as their combined measure (MVPA), may therefore help determine whether the association with dementia differs according to activity intensity.

Although several UK Biobank studies have examined physical activity and dementia risk in the general population, evidence specifically among individuals with hypertension remains limited. Our study builds upon previous UK Biobank analyses by specifically targeting adults with hypertension and incorporating both objectively measured and self-reported physical activity. In addition, we evaluated different physical activity domains and examined potential differences between early-onset and late-onset dementia. Therefore, the present study aimed to address these knowledge gaps using two complementary cohorts from the UK Biobank.

## Materials and methods

### Patient and public involvement statement

Patients and/or the public were not involved in the design, or conduct, or reporting, or dissemination plans of this research.

### Study population

The UK Biobank is a population-based prospective study that recruited more than 0.5 million participants aged 37–73 years between 2006 and 2010 from the UK. The detailed design and methods have been described previously (Sudlow et al., 2015). All participants provided written informed consent, and the study was approved by the North West Multi-center Research Ethics Committee (MREC) (REC reference: 21/NW/0157). This research has been conducted with the UK Biobank Resource under project 144894. For the present analysis, general inclusion criteria included UK Biobank participants with a clinical diagnosis of hypertension and valid consent for research data use. We first excluded 69 participants who formally withdrew their consent for UK Biobank research, as their records cannot be used for any analysis per UK Biobank data-use rules. Following this initial exclusion, we constructed two analytical sub-cohorts with additional eligibility criteria. The accelerometer-based cohort required valid wrist-worn accelerometer measurements (≥72 h of valid wear time and successful calibration) collected between 2013 and2015 and no prevalent dementia prior to accelerometer assessment. The questionnaire-based cohort required complete responses to the short International Physical Activity Questionnaire (IPAQ) collected at baseline enrolment. Participants without linkage to hospital inpatient and national death registry outcome records were further excluded from both cohorts. We examined the associations between PA and incident dementia in the above-defined accelerometer-based and questionnaire-based cohorts. The details of the study population selection process are shown in Figure 1. We analyzed data from a questionnaire-based cohort and an accelerometer-based cohort in parallel. The rationale for utilizing both cohorts is complementary: the questionnaire cohort provides highly granular data on specific PA domains (e.g., housework, leisure time), while the accelerometer cohort provides objective, device-measured physical activity intensities, thus cross-validating our findings.

**FIGURE 1:**
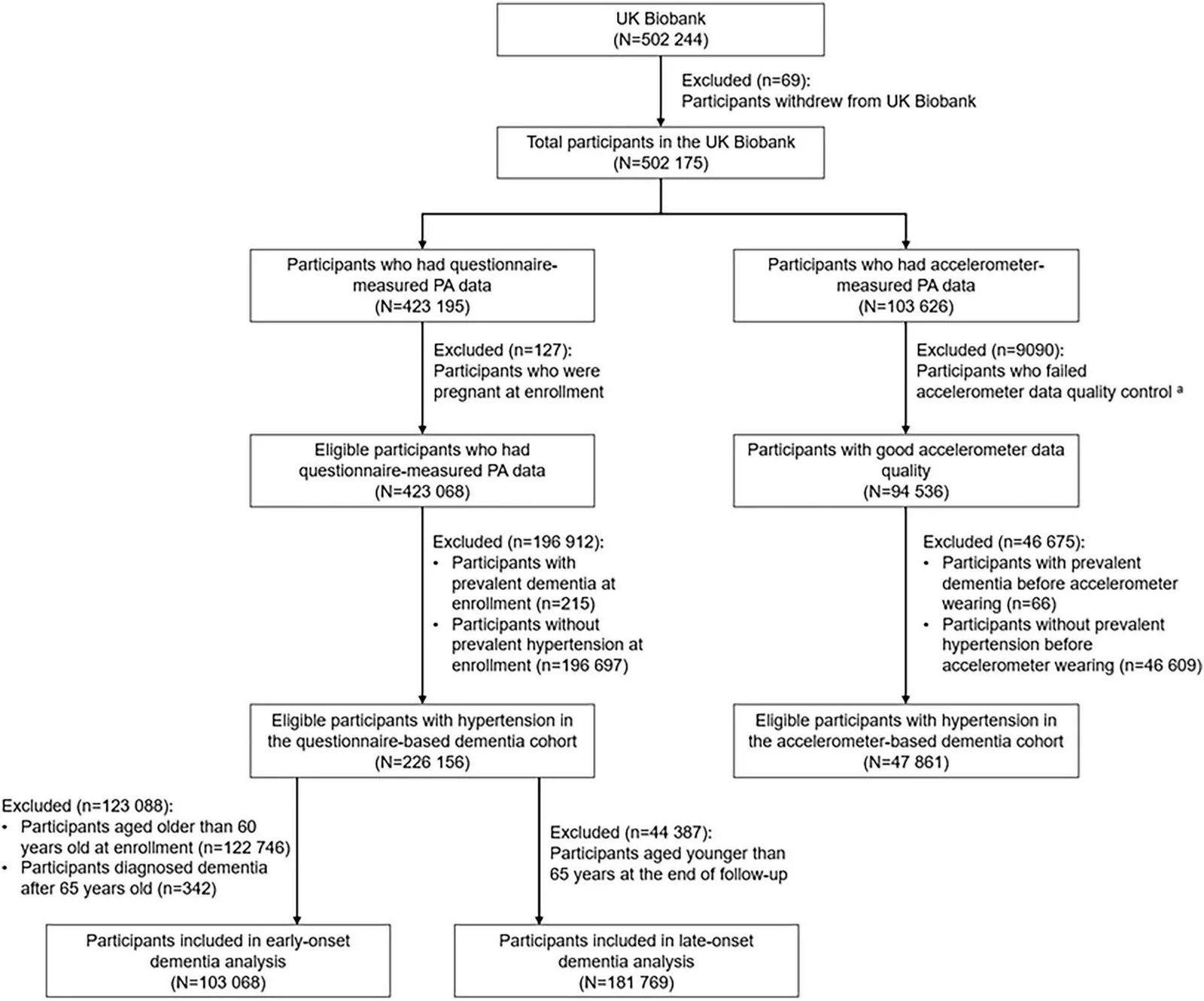
The flow chart of participants through the study. ^a^Reasons for accelerometer data quality control failure included insufficient wear time (<72 h), failed calibration, and insufficient data. PA, physical activity.

### Accelerometer-based and questionnaire-based PA assessment

To objectively measure physical activity, a wrist-worn accelerometer assessment was conducted between 2013 and 2015 (approximately 5–7 years after baseline recruitment). Importantly, the accelerometer-based cohort should be interpreted as a delayed-entry cohort rather than a true baseline exposure cohort, given the temporal gap between baseline recruitment and physical activity measurement. To minimize temporal mismatch, covariates were updated using data closest to this assessment, and participants with pre-existing dementia prior to the accelerometer wear were excluded (Doherty et al., 2017).

Participants who agreed to participate were sent an Axivity AX3 wrist-worn triaxial accelerometer and wore it for 7 days. The dominant wrist of each participant was used over 7 days at 100 Hz and a dynamic range of ±8 g (Doherty et al., 2017). The accelerometer-based PA data were recorded in milligravity units (mg). LPA, MPA, and VPA were identified as having 30–125, 125–400, and >400 mg intensity, respectively (Strain et al., 2020; Xiang et al., 2023; Doherty et al., 2017). These thresholds are derived from the standard UK Biobank accelerometer processing pipeline and have been validated in free-living populations using indirect calorimetry and energy expenditure measurements.

In the questionnaire-based cohort, each individual was assessed based on a well-validated short International Physical Activity Questionnaire (IPAQ) at recruitment (IPAQ Research Committee, 2005; Lee et al., 2011; Yoon et al., 2017). The IPAQ questionnaire collected the frequency and duration of daily walking, MPA, and VPA for at least 10 continuous minutes in a typical week. In addition, the IPAQ also collected five PA-related items (leisure time, housework, job, transportation for others, and transportation for work), and details of the questionnaire were presented in Supplementary Table 1. PA calculation and categories for both cohorts were detailed in Supplementary Method 1 (Fan et al., 2014). While questionnaire data are inherently susceptible to recall and social desirability biases, we applied standardized IPAQ scoring algorithms and adjusted for multiple lifestyle covariates to reduce systematic error. Importantly, the parallel use of accelerometer-based objective physical activity measurements in an independent cohort provides cross-validation and helps to mitigate potential recall-related bias in self-reported data.

### Ascertainment of outcome

Dementia was ascertained through hospital inpatient and death records in the UK Biobank. Data on dates and causes of death were obtained from the death registries of the National Health Service Information Centre for England and Wales, and the National Health Service Central Register for Scotland. We used the International Statistical Classification of Diseases (ICD) codes to define non-fatal and fatal dementia cases. The detailed definitions are presented in Supplementary Table 2. Dementia cases were identified via hospital inpatient and death records, which primarily capture more severe or advanced stages of cognitive decline, potentially leading to an under-detection of mild dementia cases. This ascertainment approach may result in outcome misclassification, which is expected to be non-differential with respect to physical activity and would likely bias the observed associations toward the null. Therefore, the reported associations may represent conservative estimates of the true effect. The follow-up time in the accelerometer-based and questionnaire-based cohort was calculated from the date of completion of accelerometer wear and enrollment to the first occurrence of dementia in the follow-up visits, the censoring date (31 December 2022), or death, whichever came first. In the questionnaire-based cohort, early-onset dementia was defined as dementia diagnosed before 65 years of age, and the analyses were conducted among participants aged younger than 60 years at enrollment. Late-onset dementia was defined as dementia diagnosed at 65 years or older, and the analyses were conducted among individuals aged 65 years or older at the end of follow-up.

### Assessments of covariates

Demographic covariates in this study included age, sex (male, female), ethnicity [White, non-White (Asian, Black, and other)], Townsend deprivation index, and education levels (college/university degree or above, high school degree or below). Information was collected from a touch-screen questionnaire at baseline. Lifestyle factors were based on participants’ cigarette smoking (never, previous, current), alcohol consumption, and ideal diet according to previous studies (detailed in Supplementary Table 3; Lourida et al., 2019; Amerzadeh and Takian, 2020). Healthy drinking was defined as daily consumption of less than 14 g for females and 28 g for males. Other covariates included the presence of APOE ε4, body mass index (BMI), glycated hemoglobin (HbA1c), triglyceride (TG), systolic blood pressure (SBP), apolipoprotein A, apolipoprotein B, depressive symptoms, diabetes, and parental dementia. We addressed missing data in either continuous or categorical covariates using multiple imputations by chained equations (Lee and Carlin, 2010; Liu and De, 2015). Detailed information about missing covariates was presented in Supplementary Tables 4, 5.

### Statistical analysis

We assessed the association between different types of PA and the risk of dementia using Cox proportional hazard regression models. Our model selection strategy was guided by prior literature and conceptual causal frameworks to identify potential confounders. The fully adjusted model included sex, age at enrolment, ethnicity, Townsend deprivation index, education attainment, smoking, healthy drinking, ideal diet, BMI, apolipoprotein A, apolipoprotein B, TG, HbA1c, SBP, diabetes, depression, parental dementia, and APOE ε4 carriers in the questionnaire-based cohort. We conducted analogs models to investigate the association between quartiles of the five PA categories measured in METs and the risk of dementia. Effect estimates were reported as hazard ratios (HRs) with 95% confidence intervals. For continuous PA measures, HRs were expressed per unit increase and per standard deviation increase where appropriate. For categorical analyses, comparisons were made between the highest and lowest categories to facilitate interpretation of relative risk. In addition, rate advancement periods were calculated to translate relative risk estimates into differences in chronological age to improve clinical interpretability. To estimate the impact of PA on the relation of age to dementia, we calculated the rate advancement periods (RAPs) for the five PA categories (detailed in Supplementary Method 2; Batty et al., 2020).

Additionally, we examined the associations of types and quartiles of PA with the risk of early-onset dementia and late-onset dementia only in the questionnaire-based cohort, due to the limited events of early-onset dementia in the accelerometer-based cohort. To quantify the HR differences between early-onset dementia and late-onset dementia in each of the PA items and categories, we calculated a ratio of HRs as has been done in previous studies using the UK Biobank (detailed in Supplementary Method 2; Batty et al., 2020). We performed multiple secondary analyses (detailed in Supplementary Method 3). Specifically, to minimize the risk of reverse causation, we conducted a key sensitivity analysis lagging the follow-up period by excluding participants who developed dementia or died within the first 2 years of follow-up.

Throughout these analyses, we assessed the proportionality of hazards assumption using the Schoenfeld residuals technique, and no violations were observed. All analyses were conducted using SAS 9.4 software (SAS Institute, Cary, NC) and R 4.3.2 (R Foundation for Statistical Computing, Vienna, Austria). A two-sided *P*-value < 0.05 was considered significant.

Because this was an observational study, the primary analysis focused on the association between overall physical activity and incident dementia. Analyses by physical activity domains, early- and late-onset dementia, continuous and categorical exposure models, subgroup analyses, and sensitivity analyses were considered secondary exploratory analyses rather than confirmatory hypothesis-testing analyses. Therefore, no formal adjustment for multiple comparisons was performed. These analyses were intended to generate hypotheses for future research, and the corresponding findings should be interpreted with appropriate caution.

We acknowledge that residual risk of overadjustment cannot be fully excluded, as some clinical covariates may potentially lie on the causal pathway between physical activity and dementia. However, given the observational nature of the study, residual confounding due to unmeasured or imperfectly measured factors cannot be fully excluded. Moreover, the subgroup and sensitivity analyses were exploratory and were performed to assess the robustness and potential heterogeneity of the observed associations. Accordingly, these findings should be regarded as hypothesis-generating rather than confirmatory.

## Results

### Baseline characteristics

Our study included 47,861 participants in the accelerometer-based cohort with an average age of 64.13 ± 7.17 years and 51.4% (*n* = 24,588) were male. Compared with participants with the lowest quartile of TPA, participants with the highest quartile were more likely to be younger, female, and more likely to have ideal diet, lower Townsend deprivation index, BMI, TG, HbA1c, and lower prevalence of diabetes and depression (Table 1). Similar results were also observed in baseline characteristics of the 226,156 participants involved in the questionnaire-based cohort (Supplementary Table 6). Due to the sub-sampling design of accelerometer monitoring in the UK Biobank, a subset of participants overlapped between the two cohorts; for these participants, device-measured physical activity parameters were also presented in Supplementary Table 6 for comparison. Baseline characteristics in early-onset dementia and late-onset dementia cohorts were shown in Supplementary Table 7.

**TABLE 1:** Baseline characteristics by accelerometer-measured total physical activity (*n* = 47 861).

Variables	Total (*n* = 47,861)	TPA, MET-min/week			
		Q1 (≤6817.10)	Q2 (6817.10–8239.93)	Q3 (8239.93–9713.09)	Q4 (>9713.09)
		(*n* = 11,966)	(*n* = 11,963)	(*n* = 11,967)	(*n* = 15,351)
Age, years [mean (SD)]^a^	64.13 (7.17)	65.15 (7.10)	64.54 (7.09)	64.02 (7.01)	62.82 (7.27)
Male, *n* (%)	24,588 (51.4)	7,172 (59.9)	6,304 (52.7)	5,848 (48.9)	5,264 (44.0)
White, *n* (%)	46,441 (97.0)	11,659 (97.4)	11,629 (97.2)	11,622 (97.1)	11,531 (96.4)
Townsend deprivation index [mean (SD)]	−1.77 (2.79)	−1.53 (2.96)	−1.78 (2.79)	−1.90 (2.71)	−1.87 (2.69)
College/university degree, *n* (%)	18,371 (38.4)	4,499 (37.6)	4,718 (39.4)	4,699 (39.3)	4,455 (37.2)
Accelerometer data					
**Season of accelerometer wearing, *n* (%)**					
Spring	10,666 (22.3)	2,454 (20.5)	2,616 (21.9)	2,759 (23.1)	2,837 (23.7)
Summer	12,587 (26.3)	2,767 (23.1)	3,004 (25.1)	3,245 (27.1)	3,571 (29.8)
Autumn	14,248 (29.8)	3,682 (30.8)	3,612 (30.2)	3,533 (29.5)	3,421 (28.6)
Winter	10,360 (21.6)	3,063 (25.6)	2,731 (22.8)	2,430 (20.3)	2,136 (17.9)
LPA, MET-min/week [mean (SD)]	6392.54 (1520.00)	4589.61 (868.01)	6005.98 (614.18)	6891.22 (714.98)	8083.34 (1045.04)
MPA, MET-min/week [mean (SD)]	1693.44 (882.62)	896.85 (427.85)	1404.28 (480.65)	1837.04 (565.17)	2635.58 (869.63)
VPA, MET-min/week [mean (SD)]	203.90 (291.15)	81.23 (128.33)	144.41 (178.68)	216.91 (246.94)	373.03 (427.03)
Lifestyle factors, n (%)					
**Cigarette smoking**					
Never	25,828 (54.0)	5,981 (50.0)	6,562 (54.9)	6,598 (55.1)	6,687 (55.9)
Previous	18,958 (39.6)	4,941 (41.3)	4,672 (39.1)	4,707 (39.3)	4,638 (38.8)
Current	3,075 (6.4)	1,044 (8.7)	729 (6.1)	662 (5.5)	640 (5.3)
Healthy alcohol consumption	38,914 (81.3)	9,862 (82.4)	9,773 (81.7)	9,685 (80.9)	9,594 (80.2)
Ideal diet	7,381 (15.4)	1,554 (13.0)	1,798 (15.0)	1,878 (15.7)	2,151 (18.0)
Clinical characteristics					
BMI, kg/m^2^ [mean (SD)]	27.90 (4.76)	29.47 (5.30)	28.19 (4.65)	27.41 (4.40)	26.54 (4.13)
Apolipoprotein A, g/L [mean (SD)]	1.55 (0.26)	1.49 (0.25)	1.53 (0.25)	1.57 (0.26)	1.60 (0.26)
Apolipoprotein B, g/L [mean (SD)]	1.04 (0.23)	1.03 (0.24)	1.04 (0.23)	1.04 (0.23)	1.04 (0.23)
TG, mmol/L [mean (SD)]	1.83 (1.01)	1.99 (1.06)	1.87 (1.02)	1.78 (0.99)	1.69 (0.94)
SBP, mmHg [mean (SD)]	148.66 (15.46)	148.08 (16.25)	148.42 (15.50)	148.91 (14.98)	149.21 (15.07)
HbA1c, % [mean (SD)]	5.47 (0.57)	5.56 (0.68)	5.48 (0.59)	5.44 (0.51)	5.42 (0.48)
Diabetes, *n* (%)^a^	4,422 (9.2)	1,751 (14.6)	1,128 (9.4)	843 (7.0)	700 (5.9)
Depression, *n* (%)^a^	13,796 (28.8)	3,948 (33.0)	3,438 (28.7)	3,263 (27.3)	3,147 (26.3)
Parental dementia, *n* (%)	6,251 (13.1)	1,501 (12.5)	1,622 (13.6)	1,578 (13.2)	1,550 (13.0)
APOE ε4 carriers, *n* (%)	11,132 (23.3)	2,675 (22.4)	2,791 (23.3)	2,744 (22.9)	2,922 (24.4)

^a^Measured at accelerometer wearing. BMI, body mass index; LPA, light physical activity; MET, metabolic equivalent of energy; MPA, moderate physical activity; HbA1c, glycated hemoglobin; SBP, systolic blood pressure; SD, standard deviation; TG, triglyceride; TPA, total physical activity; VPA, vigorous physical activity.

### Associations of questionnaire-based PA items with the risk of dementia

Figure 2 and Supplementary Table 8 present the associations of questionnaire-based PA items with the risk of dementia. Regarding PA in leisure time, the higher level of moderate exercise and stair climbing were associated with a lower risk of dementia. Regarding housework-related PA, the higher quartiles of light and heavy DIY demonstrate 10%–24% and 17%–25% lower risks of dementia, respectively. No significant association was observed in job-related activity and transportation for work. Regarding transportation for others, cycling was associated with a 20% lower risk of dementia. Additionally, we explored the associations of physical activity items with early-onset and late-onset dementia (Supplementary Table 9). Given the exploratory nature of these secondary analyses, these findings should be interpreted as hypothesis-generating. For more than 10 times/day of stair climbing, the HR was typically lower for early-onset dementia (HR: 0.57, 95% CI: 0.39, 0.85) than for late-onset dementia (HR: 0.88, 95% CI: 0.81, 0.96), yielding a ratio of HR less than 1 (0.65, 95% CI: 0.43, 0.97).

**FIGURE 2:**
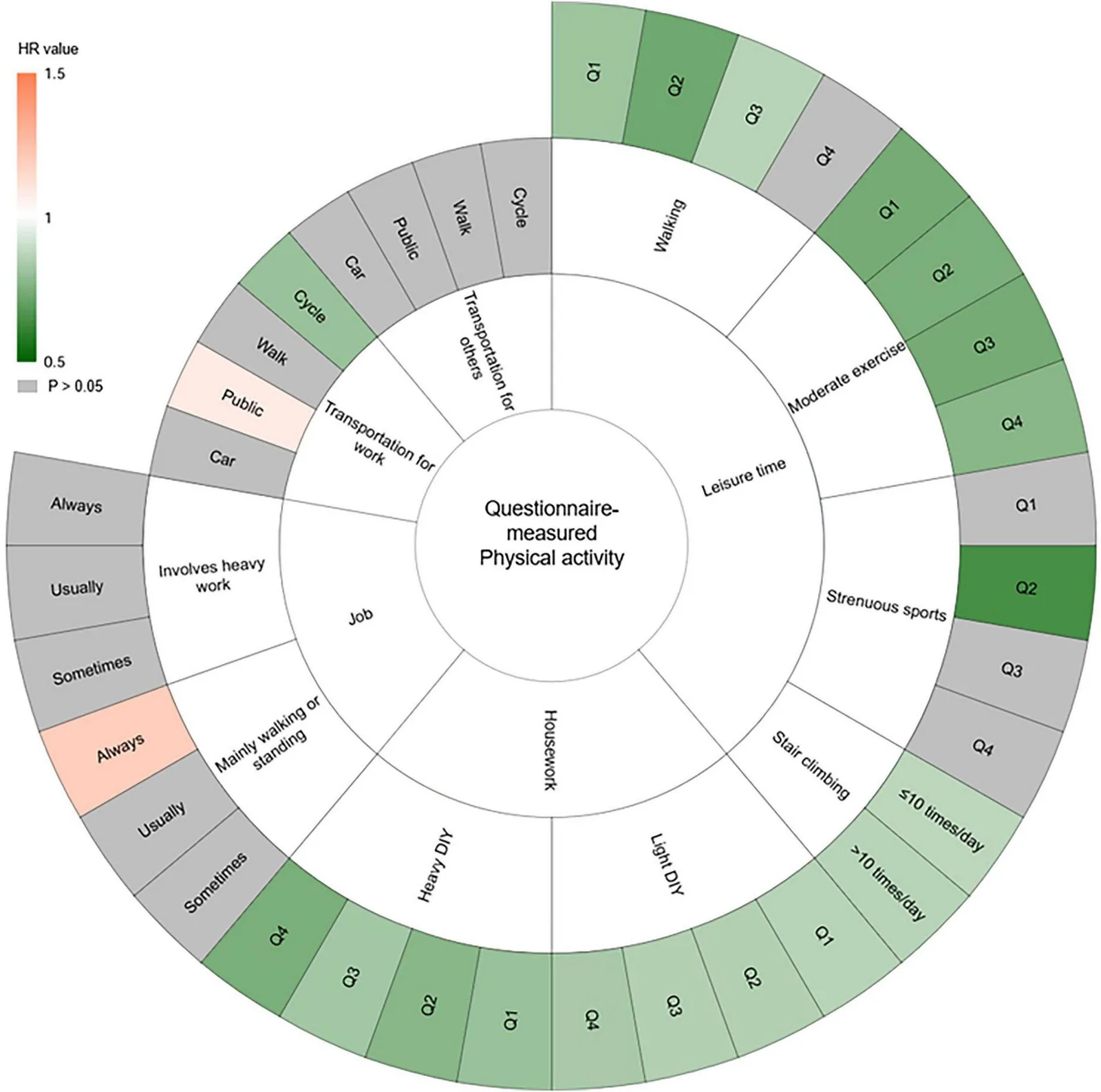
HRs for the associations of questionnaire-based physical activity items and incident dementia. A darker color in a gradient from white to red shows higher hazard ratios of dementia. A darker color in a gradient from white to green shows lower hazard ratios of dementia. Gray shows non-significant associations. Models were adjusted for sex, age at enrollment, ethnicity, Townsend deprivation index, education attainment, lifestyle factors (smoking, healthy drinking, ideal diet), clinical characteristics (BMI, apolipoprotein A, apolipoprotein B, TG, HbA1c, SBP, diabetes, depression, parental dementia, APOE ε4 carriers). DIY, do-it-yourself; HR, hazard ratio.

### Associations of PA categories with the risk of dementia

During a median of 8.1 (7.5, 8.6) years of follow-up, 574 incident dementia were observed in the accelerometer-based cohort. The absolute dementia incidence rates from the lowest (Q1) to the highest (Q4) quartiles of TPA were 1.83, 1.52, 1.39, and 1.14 cases per 1,000 person-years, respectively. Compared with the lowest quartile of MPA, the second (HR: 0.56, 95% CI: 0.45, 0.69), third (HR: 0.56, 95% CI: 0.45, 0.71) and highest quartile (HR: 0.41, 95% CI: 0.31, 0.54) were associated with lower risk of dementia (*P* for trend <0.001). Similar trends were observed for VPA, MVPA, and TPA (Figure 3). Although higher levels of moderate-to-vigorous physical activity were associated with lower relative risks of dementia, the absolute differences in incidence rates across physical activity categories were modest. In the questionnaire-based cohort, during a median of 13.7 (12.9, 14.4) years of follow-up, 5647 incident dementia were observed. The absolute dementia incidence rates across TPA quartiles (Q1–Q4) were 2.18, 1.95, 1.74, and 1.62 cases per 1,000 person-years, respectively. The associations of MPA, VPA, MVPA, and TPA with the risk of dementia were also significant, although they were attenuated compared with those observed for accelerometer-measured PA. Based on RAP analyses in the accelerometer-based cohort (Supplementary Table 10), the dementia incidence rate for the lowest quartile of MPA was equivalent to those with quartiles 2, 3, and 4 when they were 3.20, 3.17, and 4.91 years older, respectively. These findings indicate that higher physical activity levels were associated with a lower age-equivalent risk of dementia, as reflected by the RAP estimates. Furthermore, in the accelerometer-based cohort, we observed linear relationships of MPA, MVPA, TPA, and non-linear relationships of VPA. In the questionnaire-based cohort, we observed the non-linear association of MPA, VPA, and MVPA with the risk of dementia (Supplementary Figure 1).

**FIGURE 3:**
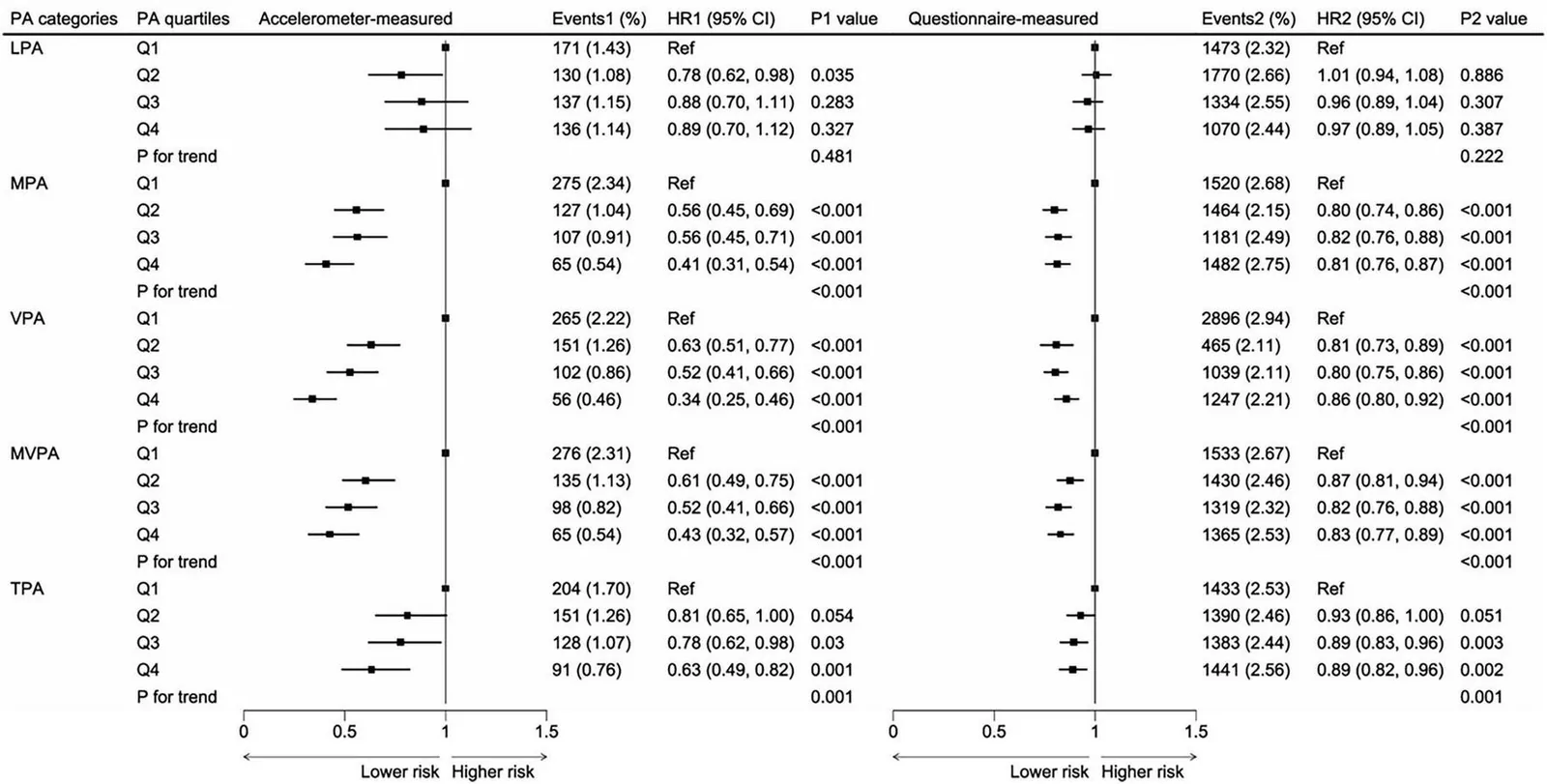
Associations between categories of accelerometer-measured physical activity or questionnaire-measured physical activity and incident dementia. Models were adjusted for sex, age, ethnicity, Townsend deprivation index, education attainment, lifestyle factors (smoking, healthy drinking, ideal diet), clinical characteristics (BMI, apolipoprotein A, apolipoprotein B, TG, HbA1c, SBP, diabetes, depression, parental dementia, APOE ε4 carriers). Event1, HR1 and P1 values refer to the events of dementia, HR and *P*-values for the associations in the accelerometer-based cohort. Event2, HR2 and P2 value refers to the events of dementia, HR and *P*-value for the associations in the questionnaire-based cohort. CI, confidence interval; HR, hazard ratio; LPA, light physical activity; MPA, moderate physical activity; PA, physical activity; Ref, reference; TPA, total physical activity; VPA, vigorous physical activity.

For the comparison of early-onset and late-onset dementia incidence (Table 2), the third and highest quartiles of MPA were associated with lower HRs for early-onset dementia (Q3: HR, 0.57; 95% CI, 0.41–0.81; Q4: HR, 0.57; 95% CI, 0.42–0.79). The corresponding HRs for late-onset dementia were higher (Q3: HR, 0.83; 95% CI, 0.77–0.89; Q4: HR, 0.85; 95% CI, 0.79–0.92). The corresponding ratios of HRs were <1 (Q3: 0.67, 95% CI, 0.47–0.95; Q4: 0.69, 95% CI, 0.50–0.96). These exploratory findings suggest that the association between MPA and dementia risk may be stronger for early-onset dementia than for late-onset dementia. However, these observations should be interpreted cautiously and require confirmation in future studies specifically designed to test these hypotheses. Furthermore, we observed non-linear relationships between MPA and MVPA and the risk of early-onset dementia and late-onset dementia (Supplementary Figure 2).

**TABLE 2:** Associations between questionnaire-measured physical activity and incident early-onset dementia and late-onset dementia.

PA categories	Early-onset dementia^a^	HR (95% CI)^b^	*P*-value	Late-onset dementia^a^	HR (95% CI)^b^	*P*-value	Ratio of HRs (95% CI)
	Events, *n* (%)			Events, *n* (%)			
LPA							
Q1	87 (0.31)	Ref	–	1,373 (2.77)	Ref	–	–
Q2	71 (0.29)	0.97 (0.71, 1.33)	0.870	1,660 (3.05)	0.99 (0.92, 1.07)	0.865	0.98 (0.71, 1.35)
Q3	68 (0.22)	0.72 (0.52, 1.00)	0.047	1,283 (2.99)	0.97 (0.90, 1.05)	0.518	0.74 (0.53, 1.03)
Q4	56 (0.27)	0.79 (0.56, 1.11)	0.179	1,006 (2.89)	0.97 (0.89, 1.05)	0.441	0.82 (0.58, 1.16)
*P* for trend	–	–	0.056	–	–	0.369	–
MPA							
Q1	113 (0.39)	Ref	–	1,560 (3.06)	Ref	–	–
Q2	64 (0.25)	0.72 (0.53, 0.98)	0.037	1,213 (2.60)	0.83 (0.77, 0.89)	<0.001	0.87 (0.64, 1.20)
Q3	48 (0.20)	0.57 (0.41, 0.81)	0.001	1,165 (2.94)	0.85 (0.79, 0.92)	<0.001	0.67 (0.47, 0.95)
Q4	57 (0.23)	0.57 (0.42, 0.79)	0.001	1384 (3.11)	0.83 (0.77, 0.90)	<0.001	0.69 (0.50, 0.96)
*P* for trend	–	–	<0.001	–	–	<0.001	–
VPA							
Q1	145 (0.34)	Ref	–	2,735 (3.36)	Ref	–	–
Q2	25 (0.25)	0.87 (0.57, 1.34)	0.525	270 (2.61)	0.87 (0.77, 0.99)	0.032	1.00 (0.64, 1.56)
Q3	58 (0.20)	0.73 (0.54, 1.00)	0.047	1,148 (2.45)	0.79 (0.74, 0.85)	<0.001	0.92 (0.67, 1.27)
Q4	54 (0.26)	0.88 (0.64, 1.22)	0.444	1,169 (2.70)	0.85 (0.80, 0.91)	<0.001	1.03 (0.74, 1.44)
*P* for trend	–	–	0.155	–	–	<0.001	–
MVPA							
Q1	104 (0.39)	Ref	–	1,601 (3.07)	Ref	–	–
Q2	65 (0.26)	0.75 (0.55, 1.02)	0.068	1,166 (2.91)	0.91 (0.85, 0.99)	0.019	0.82 (0.59, 1.13)
Q3	49 (0.19)	0.58 (0.41, 0.82)	0.002	1,263 (2.77)	0.85 (0.79, 0.92)	<0.001	0.68 (0.48, 0.97)
Q4	64 (0.25)	0.69 (0.50, 0.95)	0.023	1,292 (2.94)	0.85 (0.79, 0.91)	<0.001	0.81 (0.59, 1.13)
*P* for trend	–	–	0.006	–	–	<0.001	–
TPA							
Q1	96 (0.37)	Ref	–	1,354 (2.97)	Ref	–	–
Q2	71 (0.28)	0.84 (0.62, 1.15)	0.274	1,306 (2.88)	0.93 (0.86, 1.00)	0.053	0.91 (0.66, 1.25)
Q3	53 (0.21)	0.64 (0.45, 0.90)	0.009	1,299 (2.86)	0.89 (0.83, 0.96)	0.004	0.71 (0.50, 1.01)
Q4	62 (0.24)	0.65 (0.47, 0.90)	0.009	1,363 (3.00)	0.89 (0.83, 0.96)	0.003	0.73 (0.52, 1.02)
*P* for trend	–	–	0.003	–	–	0.002	–

^a^Early-onset dementia was defined as dementia diagnosed before 65 years of age, and the analyses were conducted among participants aged younger than 60 years at enrollment. Late-onset dementia was defined as dementia diagnosed at 65 years or older, and the analyses were conducted among individuals aged 65 years or older at the end of follow-up.

^b^Models were adjusted for sex, age at enrollment, ethnicity, Townsend deprivation index, education attainment, lifestyle factors (smoking, healthy drinking, ideal diet), clinical characteristics (BMI, apolipoprotein A, apolipoprotein B, TG, HbA1c, SBP, diabetes, depression, parental dementia, APOE ε4 carriers). CI,: confidence interval; HR, hazard ratio; LPA, light physical activity; MPA, moderate physical activity; PA, physical activity; Ref, reference; TPA, total physical activity; VPA, vigorous physical activity.

### Sensitivity analyses

Subgroup analyses showed that participants without APOE ε4 with higher quartiles of MPA had lower risk of dementia than participants with APOE ε4 (*P* for interaction = 0.045) in the accelerometer-based cohort (Supplementary Table 13). Similar significant interaction of APOE ε4 subgroups (*P* for interaction = 0.018) was observed in the questionnaire-based cohort (Supplementary Table 16). Results of subgroups were shown in Supplementary Tables 11–14 for accelerometer-based cohort and Supplementary Tables 15–18 for questionnaire-based cohort.

The findings were generally consistent across competing risk models (Supplementary Table 19), excluding participants with incomplete covariates (Supplementary Table 20). To reduce potential reverse causation, we conducted sensitivity analyses excluding participants who developed dementia or died within the first 2 years of follow-up (Supplementary Table 21) and alternative MPA threshold in the accelerometer-based cohort (Supplementary Table 22). When LPA, MPA, and VPA were simultaneously included in the same model and mutually adjusted for each other (Supplementary Table 23), the associations of MPA and VPA with lower dementia risk remained significant. In contrast, the highest quartile of LPA was associated with a 38% higher hazard rate of dementia compared with the lowest quartile in the accelerometer-based cohort (HR: 1.38, 95% CI: 1.06–1.79). However, given the observational nature of the study, residual confounding due to unmeasured or imperfectly measured factors cannot be fully excluded.

## Discussion

By leveraging a large, prospective cohort of UK adults with hypertension, we observed that higher levels of MPA, VPA, MVPA, and TPA were associated with reduced hazards of dementia in both the accelerometer- and questionnaire-based cohorts. Engaging in leisure-time and housework activities was also associated with a lower risk of incident dementia. Furthermore, we found that MPA was more strongly associated with a lower risk of early-onset dementia than late-onset dementia.

It is critical to compare the findings from our two distinct exposures. We observed that the estimated hazard ratios were consistently more pronounced in the accelerometer-based cohort than in the questionnaire-based cohort. This discrepancy is likely attributable to non-differential measurement error and inherent recall biases embedded in self-reported data, which typically attenuate true underlying associations. Conversely, wearable devices capture incidental, brief bouts of moderate-to-vigorous activity that participants often omit in self-reports. The alignment of both methods in demonstrating significant inverse associations, however, reinforces the internal validity of our findings.

Our study extends previous UK Biobank analyses by focusing specifically on adults with hypertension and comparing objective and self-reported physical activity. The evidence on the prospective association between PA and dementia has mostly been derived from the general population, and the results remain inconclusive. Two cohorts using accelerometer-measured data showed a significant trend toward a low risk of dementia with increasing PA levels (Buchman et al., 2012; Petermann-Rocha et al., 2021). Previous cohort studies have extensively examined the inverse association between self-reported PA and dementia (Sabia et al., 2017; Tan et al., 2017; Soni et al., 2019; Dupré et al., 2020), which is consistent with our findings. While other meta-analyses in healthy older adults showed a non-significant association between self-reported or device-measured PA and more than 10-year follow-up global cognitive decline and non-significant effect of aerobic exercise intervention (Kelly et al., 2014; Iso-Markku et al., 2024). It is imperative to note that dementia prevention involves an intricate interplay among multiple modifiable risk factors. Beyond physical activity, sleep quality and duration have recently emerged as cornerstone lifestyle determinants of cognitive longevity. Evidence indicates that adequate, high-quality sleep exerts powerful neuroprotective benefits by sustaining brain network integrity and enhancing functional connectivity (Pini et al., 2020), which closely mirrors the physiologic pathways hypothesized for active exercise.

Our observational data cannot directly capture biological processes; therefore, the underlying mechanisms linking physical activity to dementia remain speculative. Existing literature suggests several potential pathways that may partly explain our findings in hypertensive populations. In individuals with elevated blood pressure, physical activity has been hypothesized to improve endothelial function, reduce systemic blood pressure, and attenuate cerebrovascular white matter damage (Liang et al., 2024). Experimental and animal studies further suggest that exercise may be associated with increased neurogenesis, elevated brain-derived neurotrophic factor (BDNF) levels, and improved cerebral oxygenation (Brown et al., 2013; Leckie et al., 2014; Jensen et al., 2015; Renke et al., 2022; Beinlich et al., 2024). These proposed mechanisms may be relevant to the observed associations in individuals with hypertension. However, these biological pathways were not directly measured in the present study and should therefore be regarded as hypothetical explanations rather than evidence derived from our findings.

We found that the MPA and VPA were significantly associated with a reduced risk of dementia after considering the genetic risk and other confounders, or even mutually adjusted for each other, which was in line with previous studies (Petermann-Rocha et al., 2021; Nguyen et al., 2023). Guidelines recommend that individuals with hypertension should perform at least 150 min per week of MPA, 75 min per week of VPA, or an equivalent combination of both intensities (≥600 MET/min/week) (Whelton et al., 2018; Bull et al., 2020; National Institute for Health and Care Excellence (NICE), 2023). Xiang et al. (2023) showed the protective benefit of reaching recommended MPA and MVPA on the risk of all-cause mortality and cardiovascular outcomes in individuals with hypertension. Our study extended these findings by showing that higher accelerometer-measured MPA was linearly associated with lower risks of dementia across quartiles. Notably, 75 min per week of VPA or more conferred limited additional benefits on dementia risk or even attenuated the benefits in the questionnaire-based cohort.

Excess VPA may cause an acute reflection in BP levels, which may lead to atherosclerotic plaque rupture, acute arterial thrombosis, and adverse CV events, especially in individuals with hypertension (Börjesson et al., 2016; Stone et al., 2023). We further found that MPA conferred greater benefits than VPA in certain contexts. There is growing interest in the associations between physical activity (PA) and the risk of early- and late-onset dementia, while dementia in younger populations has been relatively overlooked (Rimkeit and McIntosh, 2017; Li et al., 2023). Our analyses provide additional public health perspectives and suggest that MPA was more strongly associated with a lower risk of early-onset dementia than VPA.

If participants had higher MPA, their risk profile aligned with individuals in the lowest quartile who were several years older, as estimated by the Rate Advancement Periods (RAP). However, this statistical estimation reflects population-level risk comparisons and should not be strictly interpreted as a clinical guarantee of delaying chronological dementia onset for an individual. The age subgroup analyses in the accelerometer-based cohort also support the findings.

We observed weak associations between LPA and dementia. LPA appeared to confer fewer benefits than MPA and VPA, consistent with another UK Biobank study reporting that MPA and VPA accounted for approximately 99% of cognitive benefits in a compositional analysis including LPA, MPA, and VPA (Hu et al., 2024). In addition, accelerometer-based LPA may overlap with sedentary behavior, while questionnaire-based LPA may be subject to greater underestimation than other activity categories (Ho et al., 2022). Collectively, these findings highlight that instead of heavy focus on VPA, prioritizing MPA and MVPA might represent a more balanced approach for individuals with hypertension to lower the risk of dementia.

It should also be emphasized that the analyses of different physical activity domains, early- and late-onset dementia, subgroup analyses, and sensitivity analyses were secondary exploratory analyses. As the present study was observational, these analyses were intended to explore potentially important associations and generate hypotheses for future research rather than provide definitive confirmatory evidence. Therefore, these findings should be interpreted cautiously and warrant validation in independent cohorts and prospective studies with prespecified hypotheses.

Our questionnaire-based analyses extended the research on the associations between various types of PA and dementia incidence, including early- and late-onset dementia. We found that participation in leisure-time PA and housework activities was associated with a lower risk of dementia in individuals with hypertension, consistent with previous evidence on leisure-time PA and dementia (Su et al., 2022). Besides, a study examined the associations between job-related PA and the risk of dementia in a general population and reported the fifth quintile of job-related PA had a 32% lower risk of dementia. Contrarily, we did not find significant associations of job-related PA (Huang et al., 2023). The inconsistent findings may be attributed to distinct study populations and different classifications on job-related PA. Stair climbing showed consistent and stronger benefits for early-onset dementia than late-onset dementia, which suggested daily PA in leisure time also had health benefits.

### Clinical implications

The role of physical activity in reducing blood pressure and promoting cardiovascular health is well-established and is reflected in current hypertension guidelines (Whelton et al., 2018; Williams et al., 2018; National Institute for Health and Care Excellence (NICE), 2023). However, evidence regarding its association with dementia risk in individuals with hypertension remains limited, and current guidelines do not explicitly address optimal physical activity types or intensities for dementia prevention. Our findings support current physical activity recommendations and provide observational evidence that may inform future intervention studies. Specifically, the observed linear relationship between increased MPA or MVPA levels and a lower risk of dementia suggests that the cognitive benefits of exercise may align with its cardiovascular advantages. Additionally, the notable potential benefits of MPA for early-onset dementia are of interest, particularly in the context of the increasing prevalence of hypertension among younger populations (Gan et al., 2003; Son et al., 2018). This underscores the importance of investigating regular MPA as a potential strategy to mitigate early cognitive decline in younger hypertensive individuals. Furthermore, our study provides preliminary insights for those unable to achieve structured activity levels, indicating that daily incidental activities, such as housework PA and stair climbing, are also linked to lower risk estimates and warrant further investigation in prospective studies.

### Limitations

Our study should be interpreted in light of the following limitations. First, potential recall bias and misclassification should be acknowledged in the questionnaire-based analysis. Second, covariates used for adjustment were collected at enrollment, which may not fully reflect the participant’s baseline characteristics at the time of accelerometer wearing. Third, regardless of accelerometer-based or questionnaire-based PA, they did not collect specific categories of PA, such as aerobic exercise, resistance training, or other fitness exercise. Fourth, this study mostly consists of Caucasian participants and the generalizability may be limited to Asians and other ethnicity.

Fifth, the observational design prevents any inference of absolute causality, and despite extensive multivariable adjustment, the risk of residual confounding remains. Crucially, underlying physical frailty, subclinical somatic diseases, or baseline variations in hypertension severity (which were not fully captured in our dataset) may confound both an individual’s capacity to engage in physical activity and their baseline risk for cognitive decline. In addition, residual confounding may persist from unmeasured lifestyle and health status factors that were not fully captured despite adjustment. Sixth, reverse causation remains a concern, as prodromal or preclinical dementia may reduce physical activity prior to diagnosis; although we performed a 2-year lag analysis, longer preclinical periods cannot be fully excluded. Physical frailty may further contribute to this pathway by simultaneously reducing physical activity and reflecting early disease processes.

Seventh, the single 7-day accelerometer assessment may not fully capture long-term physical activity patterns due to seasonal variations in weather and daylight. However, evidence from the UK Biobank indicates that such seasonal effects are remarkably small at the population level (Doherty et al., 2017). In large cohorts, these short-term fluctuations primarily introduce non-differential measurement error, which tends to conservatively attenuate the true protective associations rather than create false positives. Nonetheless, the lack of repeated, multi-season device data remains a limitation, and future multi-wave longitudinal studies are warranted to track seasonal trajectories and their precise impact on cognitive outcomes. Eighth, the biological markers were only measured at enrollment, therefore it is difficult to evaluate the potential mechanism of PA, SBP, and other modifiable risk factors with dementia. Ninth, our accelerometer-based cohort may not have sufficient power to detect its effect on early- and late-onset dementia. In addition, multiple secondary analyses were performed without formal adjustment for multiple comparisons, which may increase the risk of Type I error (false-positive findings). Because these analyses were exploratory and not designed as confirmatory hypothesis tests, the corresponding findings should be interpreted as hypothesis-generating rather than definitive evidence. Independent validation in future prospective studies with pre-specified hypotheses will be important to confirm these observations.

Finally, we were unable to perform detailed stratified analyses by age groups or hypertension severity (e.g., controlled vs. uncontrolled hypertension), which may lead to heterogeneity in the observed associations, as both aging and uncontrolled hypertension are key accelerators of neurodegeneration.

## Conclusion

Higher levels of MPA, MVPA, VPA, and TPA were associated with a lower risk of dementia in individuals with hypertension, as well as engaging in leisure time and housework-related PA. MPA showed a stronger effect on early-onset dementia. Our findings support further evaluation of physical activity interventions in hypertensive populations through randomized clinical trials.

## Data Availability

The datasets presented in this study can be found in online repositories. The names of the repository/repositories and accession number(s) can be found below: www.ukbiobank.ac.uk.

## References

[B1] Amerzadeh M. Takian A. (2020). Reducing sugar, fat, and salt for prevention and control of noncommunicable diseases (n.d.) as an adopted health policy in Iran. *Med. J. Islam. Repub. Iran* 34:136. 33437732 10.34171/mjiri.34.136 PMC7787030

[B2] Batty G. D. Gale C. R. Kivimäki M. Deary I. J. Bell S. (2020). Comparison of risk factor associations in UK biobank against representative, general population based studies with conventional response rates: Prospective cohort study and individual participant meta-analysis. *BMJ* 368:m131. 10.1136/bmj.m131 32051121 PMC7190071

[B3] Beinlich F. Asiminas A. Untiet V. Bojarowska Z. Plá V. Sigurdsson B. et al. (2024). Oxygen imaging of hypoxic pockets in the mouse cerebral cortex. *Science* 383, 1471–1478. 10.1126/science.adn1011 38547288 PMC11251491

[B4] Börjesson M. Onerup A. Lundqvist S. Dahlöf B. (2016). Physical activity and exercise lower blood pressure in individuals with hypertension: Narrative review of 27 RCTs. *Br. J. Sports Med.* 50, 356–361. 10.1136/bjsports-2015-095786 26787705

[B5] Brown B. M. Peiffer J. J. Martins R. N. (2013). Multiple effects of physical activity on molecular and cognitive signs of brain aging: Can exercise slow neurodegeneration and delay Alzheimer’s disease. *Mol. Psychiatry* 18, 864–874. 10.1038/mp.2012.162 23164816

[B6] Buchman A. S. Boyle P. A. Yu L. Shah R. C. Wilson R. S. Bennett D. A. (2012). Total daily physical activity and the risk of AD and cognitive decline in older adults. *Neurology* 78, 1323–1329. 10.1212/WNL.0b013e3182535d35 22517108 PMC3335448

[B7] Bull F. C. Al-Ansari S. S. Biddle S. Borodulin K. Buman M. P. Cardon G. et al. (2020). World health organization 2020 guidelines on physical activity and sedentary behaviour. *Br. J. Sports Med.* 54, 1451–1462. 10.1136/bjsports-2020-102955 33239350 PMC7719906

[B8] Doherty A. Jackson D. Hammerla N. Plötz T. Olivier P. Granat M. H. et al. (2017). Large scale population assessment of physical activity using wrist worn accelerometers: The UK biobank study. *PLoS ONE* 12:e0169649. 10.1371/journal.pone.0169649 28146576 PMC5287488

[B9] Dupré C. Bongue B. Helmer C. Dartigues J. F. Hupin D. Roche F. et al. (2020). Physical activity types and risk of dementia in community-dwelling older people: The three-city cohort. *BMC Geriatr.* 20:132. 10.1186/s12877-020-01538-3 32276604 PMC7146952

[B10] Fan M. Lyu J. He P. (2014). Chinese guidelines for data processing and analysis concerning the international physical activity questionnaire. *Zhonghua liu xing bing xue za zhi.* 35, 961–964. Available online at: https://pubmed.ncbi.nlm.nih.gov/25376692/25376692

[B11] Gan S. K. Loh C. Y. Seet B. (2003). Hypertension in young adults–an under-estimated problem. *Singapore Med. J.* 44, 448–452. Available online at: https://pubmed.ncbi.nlm.nih.gov/14740773/14740773

[B12] GBD 2019 Dementia Forecasting Collaborators. (2022). Estimation of the global prevalence of dementia in 2019 and forecasted prevalence in 2050: An analysis for the global burden of disease study 2019. *Lancet Public Health* 7, e105–e125. 10.1016/S2468-2667(21)00249-8 34998485 PMC8810394

[B13] Gorelick P. B. Scuteri A. Black S. E. Decarli C. Greenberg S. M. Iadecola C. et al. (2011). Vascular contributions to cognitive impairment and dementia: A statement for healthcare professionals from the American heart association/american stroke association. *Stroke* 42, 2672–2713. 10.1161/STR.0b013e3182299496 21778438 PMC3778669

[B14] Ho F. K. Zhou Z. Petermann-Rocha F. Para-Soto S. Boonpor J. Welsh P. et al. (2022). Association between device-measured physical activity and incident heart failure: A prospective cohort study of 94 739 UK biobank participants. *Circulation* 146, 883–891. 10.1161/CIRCULATIONAHA.122.059663 36036153

[B15] Hogan D. B. Bailey P. Black S. Carswell A. Chertkow H. Clarke B. et al. (2008). Diagnosis and treatment of dementia: 5. Nonpharmacologic and pharmacologic therapy for mild to moderate dementia. *CMAJ* 179, 1019–1026. 10.1503/cmaj.081103 18981443 PMC2572644

[B16] Hu M. Zhang K. Su K. J. Qin T. Shen H. Deng H. W. (2024). Unveiling the link between physical activity levels and dementia risk: Insights from the UK biobank study. *Psychiatry Res.* 336:115875. 10.1016/j.psychres.2024.115875 38603980 PMC11090404

[B17] Huang X. Tan C. S. Kandiah N. Hilal S. (2023). Association of physical activity with dementia and cognitive decline in UK biobank. *Alzheimers Dementia* 15:e12476. 10.1002/dad2.12476 37671035 PMC10476274

[B18] IPAQ Research Committee. (2005). *Guidelines for Data Processing Analysis of the International Physical Activity Questionnaire (IPAQ) - Short and Long Forms.* Geneva: IPAQ Research Committee.

[B19] Iso-Markku P. Aaltonen S. Kujala U. M. Halme H. L. Phipps D. Knittle K. et al. (2024). Physical activity and cognitive decline among older adults: A systematic review and meta-analysis. *JAMA Netw. Open* 7:e2354285. 10.1001/jamanetworkopen.2023.54285 38300618 PMC10835510

[B20] Iso-Markku P. Kujala U. M. Knittle K. Polet J. Vuoksimaa E. Waller K. (2022). Physical activity as a protective factor for dementia and Alzheimer’s disease: Systematic review, meta-analysis and quality assessment of cohort and case-control studies. *Br. J. Sports Med.* 56, 701–709. 10.1136/bjsports-2021-104981 35301183 PMC9163715

[B21] Jensen C. S. Hasselbalch S. G. Waldemar G. Simonsen A. H. (2015). Biochemical markers of physical exercise on mild cognitive impairment and dementia: Systematic review and perspectives. *Front. Neurol.* 6:187. 10.3389/fneur.2015.00187 26379621 PMC4549649

[B22] Kelly M. E. Loughrey D. Lawlor B. A. Robertson I. H. Walsh C. Brennan S. (2014). The impact of exercise on the cognitive functioning of healthy older adults: A systematic review and meta-analysis. *Ageing Res. Rev.* 16, 12–31. 10.1016/j.arr.2014.05.002 24862109

[B23] Leckie R. L. Oberlin L. E. Voss M. W. Prakash R. S. Szabo-Reed A. Chaddock-Heyman L. et al. (2014). BDNF mediates improvements in executive function following a 1-year exercise intervention. *Front. Hum. Neurosci.* 8:985. 10.3389/fnhum.2014.00985 25566019 PMC4263078

[B24] Lee K. J. Carlin J. B. (2010). Multiple imputation for missing data: Fully conditional specification versus multivariate normal imputation. *Am. J. Epidemiol.* 171, 624–632. 10.1093/aje/kwp425 20106935

[B25] Lee P. H. Macfarlane D. J. Lam T. H. Stewart S. M. (2011). Validity of the international physical activity questionnaire short Form (IPAQ-SF): A systematic review. *Int. J. Behav. Nutr. Phys. Act.* 8:115. 10.1186/1479-5868-8-115 22018588 PMC3214824

[B26] Li R. Li R. Xie J. Chen J. Liu S. Pan A. et al. (2023). Associations of socioeconomic status and healthy lifestyle with incident early-onset and late-onset dementia: A prospective cohort study. *Lancet Healthy Longev.* 4, e693–e702. 10.1016/S2666-7568(23)00211-8 38042162

[B27] Liang C. Song Z. Yao X. Xiao Q. Fu H. Tang L. (2024). Exercise interventions for the effect of endothelial function in hypertensive patients: A systematic review and meta-analysis. *J. Clin. Hypertens.* 26, 599–614. 10.1111/jch.14818 38708922 PMC11180684

[B28] Liu Y. De A. (2015). Multiple imputation by fully conditional specification for dealing with missing data in a large epidemiologic study. *Int. J. Stat. Med. Res.* 4, 287–295. 10.6000/1929-6029.2015.04.03.7 27429686 PMC4945131

[B29] Livingston G. Sommerlad A. Orgeta V. Costafreda S. G. Huntley J. Ames D. et al. (2017). Dementia prevention, intervention, and care. *Lancet* 390, 2673–2734. 10.1016/S0140-6736(17)31363-6 28735855

[B30] Lourida I. Hannon E. Littlejohns T. J. Langa K. M. Hyppönen E. Kuzma E. et al. (2019). Association of lifestyle and genetic risk with incidence of dementia. *JAMA* 322, 430–437. 10.1001/jama.2019.9879 31302669 PMC6628594

[B31] Meschia J. F. Bushnell C. Boden-Albala B. Braun L. T. Bravata D. M. Chaturvedi S. et al. (2014). Guidelines for the primary prevention of stroke: A statement for healthcare professionals from the American heart association/American stroke association. *Stroke* 45, 3754–3832. 10.1161/STR.0000000000000046 25355838 PMC5020564

[B32] National Institute for Health and Care Excellence (NICE). (2023). *Hypertension in Adults: Diagnosis And Management.* London: National Institute for Health and Care Excellence.

[B33] Nguyen S. LaCroix A. Z. Hayden K. M. Di C. Palta P. Stefanick M. L. et al. (2023). Accelerometer-measured physical activity and sitting with incident mild cognitive impairment or probable dementia among older women. *Alzheimers Dementia* 19, 3041–3054. 10.1002/alz.12908 36695426 PMC10366337

[B34] Norton S. Matthews F. E. Barnes D. E. Yaffe K. Brayne C. (2014). Potential for primary prevention of Alzheimer’s disease: An analysis of population-based data. *Lancet Neurol.* 13, 788–794. 10.1016/S1474-4422(14)70136-X 25030513

[B35] Oh E. S. Rabins P. V. (2019). Dementia. *Ann. Intern. Med.* 171, ITC33–ITC48. 10.7326/AITC201909030 31476229

[B36] Petermann-Rocha F. Lyall D. M. Gray S. R. Gill J. Sattar N. Welsh P. et al. (2021). Dose-response association between device-measured physical activity and incident dementia: A prospective study from UK Biobank. *BMC Med.* 19:305. 10.1186/s12916-021-02172-5 34852818 PMC8638378

[B37] Pini L. Wennberg A. Mitolo M. Meneghello F. Burgio F. Semenza C. et al. (2020). Quality of sleep predicts increased frontoparietal network connectivity in patients with mild cognitive impairment. *Neurobiol. Aging.* 95, 205–213. 10.1016/j.neurobiolaging.2020.07.026 32846276

[B38] Renke M. B. Marcinkowska A. B. Kujach S. Winklewski P. J. (2022). A systematic review of the impact of physical exercise-induced increased resting cerebral blood flow on cognitive functions. *Front. Aging. Neurosci.* 14:803332. 10.3389/fnagi.2022.803332 35237146 PMC8882971

[B39] Rimkeit S. McIntosh J. (2017). Experiencing place: Younger people with dementia facing aged care. *Australas Psychiatry* 25, 554–561. 10.1177/1039856217706821 28488889

[B40] Sabia S. Dugravot A. Dartigues J. F. Abell J. Elbaz A. Kivimäki M. et al. (2017). Physical activity, cognitive decline, and risk of dementia: 28 year follow-up of whitehall II cohort study. *BMJ* 357, j2709. 10.1136/bmj.j2709 28642251 PMC5480222

[B41] Sierra C. Coca A. Schiffrin E. L. (2011). Vascular mechanisms in the pathogenesis of stroke. *Curr. Hypertens. Rep.* 13, 200–207. 10.1007/s11906-011-0195-x 21331606

[B42] Son J. S. Choi S. Kim K. Kim S. M. Choi D. Lee G. et al. (2018). Association of blood pressure classification in Korean young adults according to the 2017 American college of cardiology/American heart association guidelines with subsequent cardiovascular disease events. *JAMA* 320, 1783–1792. 10.1001/jama.2018.16501 30398603 PMC6248107

[B43] Soni M. Orrell M. Bandelow S. Steptoe A. Rafnsson S. d’Orsi E. et al. (2019). Physical activity pre- and post-dementia: English longitudinal study of ageing. *Aging. Ment. Health* 23, 15–21. 10.1080/13607863.2017.1390731 29039993

[B44] Stone P. H. Libby P. Boden W. E. (2023). Fundamental pathobiology of coronary atherosclerosis and clinical implications for chronic ischemic heart disease management-the plaque hypothesis: A narrative review. *JAMA Cardiol.* 8, 192–201. 10.1001/jamacardio.2022.3926 36515941 PMC11016334

[B45] Strain T. Wijndaele K. Dempsey P. C. Sharp S. J. Pearce M. Jeon J. et al. (2020). Wearable-device-measured physical activity and future health risk. *Nat. Med.* 26, 1385–1391. 10.1038/s41591-020-1012-3 32807930 PMC7116559

[B46] Su S. Shi L. Zheng Y. Sun Y. Huang X. Zhang A. et al. (2022). Leisure activities and the risk of dementia: A systematic review and meta-analysis. *Neurology* 99, e1651–e1663. 10.1212/WNL.0000000000200929 35948447 PMC9559944

[B47] Sudlow C. Gallacher J. Allen N. Beral V. Burton P. Danesh J. et al. (2015). UK biobank: An open access resource for identifying the causes of a wide range of complex diseases of middle and old age. *PLoS Med.* 12:e1001779. 10.1371/journal.pmed.1001779 25826379 PMC4380465

[B48] Tan Z. S. Spartano N. L. Beiser A. S. DeCarli C. Auerbach S. H. Vasan R. S. et al. (2017). Physical activity, brain volume, and dementia risk: The framingham study. *J. Gerontol. A Biol. Sci. Med. Sci.* 72, 789–795. 10.1093/gerona/glw130 27422439 PMC6075525

[B49] Whelton P. K. Carey R. M. Aronow W. S. Casey D. E. Jr. Collins K. J. Dennison Himmelfarb C. et al. (2018). 2017 ACC/AHA/AAPA/ABC/ACPM/AGS/APhA/ASH/ASPC/NMA/PCNA guideline for the prevention, detection, evaluation, and management of high blood pressure in adults: A report of the American college of cardiology/American heart association task force on clinical practice guidelines. *Hypertension* 71, e13–e115. 10.1161/HYP.0000000000000065 29133356

[B50] Williams B. Mancia G. Spiering W. Agabiti Rosei E. Azizi M. Burnier M. et al. (2018). 2018 ESC/ESH guidelines for the management of arterial hypertension. *Eur. Heart J.* 39, 3021–3104. 10.1093/eurheartj/ehy339 30165516

[B51] Xiang B. Zhou Y. Wu X. Zhou X. (2023). Association of device-measured physical activity with cardiovascular outcomes in individuals with hypertension. *Hypertension* 80, 2455–2463. 10.1161/HYPERTENSIONAHA.123.21663 37667966

[B52] Yoon S. J. Suh S. Y. Lee Y. J. Park J. Hwang S. Lee S. S. et al. (2017). Prospective validation of objective prognostic score for advanced cancer inpatients in South Korea: A multicenter study. *J. Palliat. Med* 20, 65–68. 10.1089/jpm.2016.0044 27898288

