# Supplementary material for: Association between physical activity and incident dementia among individuals with hypertension
Source: Front Aging Neurosci. 2026 Sep 15;18:1833102. doi: 10.3389/fnagi.2026.1833102 (PMC13619819; doi:10.3389/fnagi.2026.1833102)
Supplement: Supplementary file 1 [file Table_1.docx]

**Supplementary Methods:**

**Method 1 Accelerometer-based and Questionnaire-based PA assessment**

For accelerometer-based PA assessment, we included 5 PA categories, including light-intensity PA (LPA), moderate-intensity PA (MPA), vigorous-intensity PA (VPA), moderate to vigorous-intensity PA (MVPA), and total PA. Then, each PA category was converted to metabolic equivalent of energy (MET) (MET/min/week) as follow: LPA * 3.3, MPA * 4 and VPA * 8.^[1]^ Total PA (TPA) was calculated as the sum of LPA, MPA, and VPA, whilst MVPA was calculated as the sum between MPA and VPA. For questionnaire-based PA assessment, we calculated the minutes of LPA (i.e., walking), MPA, and VPA by multiplying the weekly frequency and duration and converted them into MET using the same algorithm. Participants were asked about their attendance (yes or no), frequency (days per week), and duration (minutes per day) for specific PA in the leisure time-related PA and housework-related PA. Similarly, we converted them into MET. For housework-related PA, the minutes of heavy do-it-yourself (DIY) activity per week were multiply six considering the mixed nature of MPA and VPA. The other three PA-related items (i.e., job, transportation for others, and transportation for work) were only reported frequency or their attendance (yes, no).

**Method 2 Rate advancement periods and ratio of HR**

**Rate advancement periods**

Rate advancement periods (RAP) describes the time period by which the rate of dementia is advanced among exposed subjects.^[2]^ In another words, it is the number of additional chronologic years that would be required to yield the equivalent risk rate for dementia incidence among the other PA levels compared to the reference PA level.^[2]^ In this study, if the number is negative, for example ‘-1’, it means that the dementia incidence rate estimated for the least active participants was equivalent to those with higher levels of PA with one years older. To calculate RAPs, we divided the logarithm coefficient (HR) for the incidence for the MVPA categories referent to participants who had the lowest quartiles of PA for the incidence associated with each yearly increase in age, e.g. log (HR_PA_) / log (HR_Age_). We used SAS ‘RAPESTIM’ macro to estimate the point and 95% CI of RAP.^[3]^

**Ratio of HR**

To quantify the difference between the hazard ratios of early-onset dementia (EOD) and late-onset dementia (LOD) with PA, we calculated a ratio of the hazard ratio (RHR) as has been published elsewhere.^[4, 5]^ The equations were as follows:

RHR =$e^{\left( ln\left( {HR}_{EOD} \right)-ln({HR}_{LOD}) \right)}$

RHR 95% CI =$e^{\left( ln\left( {HR}_{EOD} \right)-ln({HR}_{LOD}) \right)\pm\left( 1.96\times\sqrt{\left( \left( \frac{ln\left( U_{EOD} \right)-ln\left( L_{EOD} \right)}{2\times1.96} \right)^{2}+\left( \frac{ln\left( U_{LOD} \right)-ln\left( L_{LOD} \right)}{2\times1.96} \right)^{2} \right)} \right)}$

HR_EOD_, the HRs for EOD with PA; HR_LOD_, the HRs for LOD with PA; U_EOD_, the upper confidence limit of the HRs for EOD with PA; L_EOD_, the lower confidence limit of the HRs for EOD with PA; U_LOD_, the upper confidence limit of the HRs for LOD with PA; L_LOD_, the lower confidence limit of the HRs for LOD with PA.

**Method 3** **Secondary analyses**

**RCS plotting**

We assessed the nonlinear associations between PA categories and the risk of dementia using the restricted cubic spline models. To minimize the influence of outliers, we trimmed observations below the 5th percentile and above the 95th percentile based on the distribution of the PA. We specified the knots at the 25th, 50th, and 75th centiles, which were utilized for categorizing the LPA, MPA, VPA, MVPA, and TPA.

**Subgroup analyses**

We performed several subgroup analyses by age (<65 years old, ≥65 years old), sex (female, male), the presence of APOE ε4 (yes, no), years since hypertension diagnosis (<10 years, ≥10 years) and baseline SBP (<160 mmHg, ≥160 mmHg). SBP subgroup analyses were only conducted in the questionnaire-based cohort because SBP was only measured at enrollment. Age subgroup analyses were only conducted in the accelerometer-based cohort avoiding redundancy.

**Sensitivity analyses**

We performed several sensitivity analyses on the associations between PA level and the risk of dementia to ensure the robustness of our results. First, considering death as a competing event for dementia, we employed the Fine-Gray hazards using the competing risk model to examine the associations. Second, we include LPA, MPA, and VPA in one model to mutually adjust for each other. Third, to minimize potential bias from missing covariates, we conducted the main analyses among participants with complete covariate data. Fourth, to avoid potential reverse causality, we exclude participants within two years of dementia or death onset. Fifth, previous studies also used the threshold of 100 mg to differentiate LPA and MPA in accelerometer-measured data. We used the alternative threshold to define LPA and MPA as the times spent on 30 to 100, and 100 to 400 intensity activity over a week, respectively.

**Supplementary Tables:**

**Table S1. Details of questionnaire-measured physical activity-related items**

| Items | Questions | Data codes |
| --- | --- | --- |
| Leisure time |  |  |
| Strenuous sports | How many times in the last 4 weeks did you do strenuous sports? | 991 |
|  | Each time you did strenuous sports, about how long did you spend doing it? | 1001 |
| Moderate exercise | How many times in the last 4 weeks did you do other exercises such as swimming, cycling, keep fit? | 3637 |
|  | Each time you did other exercises such as swimming, cycling, keep fit, about how long did you spend doing them? | 3647 |
| Walking | How many times in the last 4 weeks did you go walking for pleasure? | 971 |
|  | Each time you went walking for pleasure, about how long did you spend doing it? | 981 |
| Stair climbing | At home, during the last 4 weeks, about how many times a DAY do you climb a flight of stairs? | 943 |
| Housework |  |  |
| Heavy DIY | How many times in the last 4 weeks did you do heavy DIY? | 2624 |
|  | Each time you did heavy DIY, about how long did you spend doing it? | 2634 |
| Light DIY | How many times in the last 4 weeks did you do light DIY? | 1011 |
|  | Each time you did light DIY, about how long did you spend doing it? | 1021 |
| Job |  |  |
| Involves heavy work | Does your work involve heavy manual or physical work? | 816 |
| Mainly walking or standing | Does your work involve walking or standing for most of the time? | 806 |
| Transportation for others |  |  |
|  | In the last 4 weeks, which forms of transport have you used most often to get about? (Not including any journeys to and from work) | 6162 |
| Transportation for work |  |  |
|  | What types of transport do you use to get to and from work? | 6143 |

**Abbreviations:** DIY: do-it-yourself.

**Table S2. Methods for disease definition**

| Disease | Data fields | Field names | Data codes | Data code definitions |
| --- | --- | --- | --- | --- |
| Dementia | 130836  130838  130840  130842 | First occurrences | - | Dementia in Alzheimer’s disease, vascular dementia, dementia in other diseases classified elsewhere, unspecified dementia |
| Dementia | 20002 | Non-cancer illness code, self-reported | 1263 | Dementia |
| Dementia | 20003 | Medications, self-reported | 1141182732, 1141150834, 1141171578, 1141167690 | Prescriptions including memantine, donepezil, galantamine and rivastigmine |
| Dementia | 41270 | Diagnoses – ICD 10 | F00, G30  F010, F011, F012, F013, F018, F019, I673  F020, G310  A810, F021, F022, F023, F024, F028, F03, F051, F106, G311, G318 | Dementia in Alzheimer's disease, Alzheimer's disease, Vascular dementia of acute onset, Multi-infarct dementia, Subcortical vascular dementia, Other vascular dementia, Vascular dementia, unspecified, Progressive vascular leukoencephalopathy, Dementia in Pick's disease, Circumscribed brain atrophy, Creutzfeldt-Jakob disease, Dementia in Creutzfeldt-Jakob disease, Dementia in Huntington's disease, Dementia in Parkinson's disease, Dementia in human immunodeficiency virus [HIV] disease, Dementia in other specified diseases classified elsewhere, Unspecified dementia, Delirium superimposed on dementia, Amnesic syndrome, Senile degeneration of brain, not elsewhere classified, Other specified degenerative diseases of nervous system |
| Dementia | 41271 | Diagnoses – ICD 9 | 3310  2904  3311  2902, 2903, 2912, 2941, 3312, 3315 | Alzheimer's disease, Arteriosclerotic dementia, Pick's disease, Senile dementia, depressed or paranoid type, Senile dementia with acute confusional state, Other alcoholic dementia, Dementia in conditions classified elsewhere, Senile degeneration of brain, Jakob-creutzfeldt disease |
| Hypertension | 4079  4080 | Diastolic blood pressure, automated reading, Systolic blood pressure, automated reading | - | Blood pressure, automated reading, diastolic and systolic. Two measures of blood pressure were taken a few moments apart.  Range returned by the Omron device is is 0-255 |
| Hypertension | 6150 | Vascular/heart problems diagnosed by doctor | - | Self-reported hypertension |
| Hypertension | 20002 | Non-cancer illness code, self-reported | 1065, 1072 | Hypertension, essential hypertension |
| Hypertension | 6153  6177 | Medication for cholesterol, blood pressure, diabetes, or take exogenous hormones, Medication for cholesterol, blood pressure or diabetes | - | Anti-hypertensive medications |
| Hypertension | 131286 | First occurrences | - | Hypertension episode, recurrent hypertension episode |
| Hypertension | 41270 | Diagnoses – ICD 10 | I10 | Essential (primary) hypertension |
| Hypertension | 41271 | Diagnoses – ICD 9 | 401 | Essential hypertension |

**Abbreviations:** ICD: International Classification of Diseases.

**Table S3. Diet component definitions used in the UK Biobank study.**

| Diet component | Intake goal | Field IDs | Amount per serving |
| --- | --- | --- | --- |
| Fruit | ≥3 servings/day | 1309 (pieces fresh fruit/day) | 1309 - 1 piece |
|  |  | 1319 (pieces dried fruit/day) | 1319 - 5 pieces |
| Vegetable | ≥3 servings/day | 1289 (tablespoons cooked vegetables/day) | 3 heaped tablespoons |
|  |  | 1299 (salad/raw vegetables/day) |  |
| (Shell) fish | ≥2 servings/week | 1329 (oily fish/week) | Once/week |
|  |  | 1339 (non-oily fish/week |  |
| Red meat | ≤2 servings/week | 1359 (poultry/week or day) | 1359-1389 – once/week |
|  |  | 1369 (beef/week or day) |  |
|  |  | 1379 (lamb or mutton/week or day) |  |
|  |  | 1389 (pork/week or day) |  |
| Processed meat | ≤1 servings/week | 1349 (processed meat/week or daily) | 1349 – 1 piece/day |
| Whole grains | ≥3 servings/day | 1438, 1448 (whole meal/wholegrain bread slices/week) | 1438/1448 – 1 slice/day |
|  |  | 1458, 1468 (bran/oat/muesli cereal bowls/week) | 1458/1468 – 1 bowl/day |
| Refined grains | ≤2 servings/week | 1438, 1448 (white/brown/other/week) | 1438/1448 – 1 slice/day |
|  |  | 1458, 1468 (biscuit/other cereal bowls/week) | 1458/1468 – 1 bowl/day |
| Sugar-sweetened  beverages | Don’t drink | 6144 (never consumes drinks containing sugar) | Only 0 servings were possible here |
| Dairy | ≥2 servings/week | 1408, 1418 | Once/week |
| Vegetable oil | Mainly used | 2654 (Benecol/Soft margarine/olive/ sunflower/other low fat oil) | Mainly used |

Field IDs and serving sizes used per diet component in UK Biobank with available data from the general baseline questionnaire. If participants achieved the intake goal they were considered to have an adequate intake of the diet component. Adequate intake of at least half of all diet components was considered ideal, and less than half was considered a poor diet.

**Table S4 The percentages of participants with missing covariates involved in accelerometer-measured physical activity analysis (n=47 861).**

| Variables | N | % |
| --- | --- | --- |
| Townsend deprivation index | 48 | 0.1 |
| Education attainment | 240 | 0.5 |
| Ethnicity | 157 | 0.33 |
| Smoking | 136 | 0.28 |
| BMI | 116 | 0.24 |
| Apolipoprotein A | 6770 | 14.15 |
| Apolipoprotein B | 3027 | 6.32 |
| TG | 2842 | 5.94 |
| HbA1c | 3062 | 6.4 |
| SBP | 1415 | 2.96 |
| APOE ε4 carriers | 1001 | 2.09 |

**Abbreviations:** BMI: body mass index; HbA1c: glycated hemoglobin; SBP: systolic blood pressure; TG: triglyceride.

**Table S5 The percentages of participants with missing covariates involved in questionnaire-measured physical activity analysis (n=226 156).**

| Variables | N | % |
| --- | --- | --- |
| Townsend deprivation index | 276 | 0.12 |
| Education attainment | 2010 | 0.89 |
| Ethnicity | 738 | 0.33 |
| Smoking | 743 | 0.33 |
| BMI | 999 | 0.44 |
| Apolipoprotein A | 31856 | 14.09 |
| Apolipoprotein B | 14329 | 6.34 |
| TG | 13301 | 5.88 |
| HbA1c | 14471 | 6.4 |
| SBP | 7014 | 3.1 |
| APOE ε4 carriers | 4717 | 2.09 |
| Strenuous sports in leisure time | 223 | 0.1 |
| Moderate exercise in leisure time | 1181 | 0.52 |
| Walking in leisure time | 669 | 0.3 |
| Stair climbing in leisure time | 785 | 0.35 |
| Heavy DIY | 6846 | 3.03 |
| Light DIY | 3936 | 1.74 |
| Job involves heavy work | 113235 | 50.07 |
| Job involves mainly walking or standing | 113267 | 50.08 |
| Transportation for others by car | 57 | 0.03 |
| Transportation for others by public | 57 | 0.03 |
| Transportation for others by walk | 57 | 0.03 |
| Transportation for others by cycle | 57 | 0.03 |
| Transportation for work by car | 121604 | 53.77 |
| Transportation for work by public | 121604 | 53.77 |
| Transportation for work by walk | 121604 | 53.77 |
| Transportation for work by cycle | 121604 | 53.77 |

**Abbreviations:** BMI: body mass index; DIY: do-it-yourself; HbA1c: glycated hemoglobin; SBP: systolic blood pressure; TG: triglyceride.

**Table S6. Baseline characteristics by questionnaire-measured total physical activity (n=226 156)**

| Variables | Total  (n=226 156) | TPA, MET-min/week |  |  |  |
| --- | --- | --- | --- | --- | --- |
|  |  | Q1 (<=762)  (n=56 586) | Q2 (763-1710)  (n=56 498) | Q3 (1711-3573)  (n=56 704) | Q4 (>3573)  (n=56 368) |
| Age, years (mean (SD)) | 58.68 (7.44) | 58.20 (7.37) | 58.63 (7.40) | 58.86 (7.46) | 59.01 (7.48) |
| Male, n (%) | 122 099 (54.0) | 29 999 (53.0) | 29 449 (52.1) | 30 401 (53.6) | 32 250 (57.2) |
| White, n (%) | 214 733 (94.9) | 53 210 (94.0) | 53 533 (94.8) | 54 018 (95.3) | 53 972 (95.7) |
| Townsend deprivation index (mean (SD)) | -1.37 (3.06) | -1.25 (3.15) | -1.41 (3.04) | -1.50 (2.99) | -1.31 (3.04) |
| College/university degree, n (%) | 67 970 (30.1) | 17 411 (30.8) | 19 156 (33.9) | 18 341 (32.3) | 13 062 (23.2) |
| Accelerometer data |  |  |  |  |  |
| Season of accelerometer wearing, n (%) |  |  |  |  |  |
| Spring | 10 145 (22.5) | 2446 (22.7) | 2711 (22.8) | 2728 (22.4) | 2260 (21.8) |
| Summer | 11 979 (26.5) | 2824 (26.3) | 3151 (26.6) | 3243 (26.7) | 2761 (26.6) |
| Autumn | 13 313 (29.5) | 3189 (29.6) | 3500 (29.5) | 3561 (29.3) | 3063 (29.5) |
| Winter | 9709 (21.5) | 2299 (21.4) | 2505 (21.1) | 2621 (21.6) | 2284 (22.0) |
| LPA, MET-min/week (mean (SD)) | 1216.76 (1732.72) | 262.76 (190.55) | 627.38 (364.46) | 1051.76 (682.39) | 2931.19 (2679.42) |
| MPA, MET-min/week (mean (SD)) | 1085.17 (1840.35) | 90.49 (122.79) | 353.69 (298.11) | 839.36 (610.21) | 3064.13 (2757.42) |
| VPA, MET-min/week (mean (SD)) | 682.70 (1599.57) | 34.99 (88.51) | 230.17 (301.63) | 618.92 (625.18) | 1850.65 (2789.04) |
| Lifestyle factors, n (%) |  |  |  |  |  |
| Cigarette smoking |  |  |  |  |  |
| Never | 118 454 (52.4) | 29 013 (51.3) | 30 284 (53.6) | 30 222 (53.3) | 28 935 (51.3) |
| Previous | 86 327 (38.2) | 21 262 (37.6) | 21 322 (37.7) | 21 905 (38.6) | 21 838 (38.7) |
| Current | 21 375 (9.5) | 6311 (11.2) | 4892 (8.7) | 4577 (8.1) | 5595 (9.9) |
| Healthy alcohol consumption | 181 758 (80.4) | 46 139 (81.5) | 45 852 (81.2) | 45 381 (80.0) | 44 386 (78.7) |
| Ideal diet | 34 169 (15.1) | 6188 (10.9) | 7904 (14.0) | 9633 (17.0) | 10 444 (18.5) |
| Clinical characteristics |  |  |  |  |  |
| BMI, kg/m^2^ (mean (SD)) | 28.43 (4.86) | 29.59 (5.45) | 28.40 (4.79) | 27.92 (4.51) | 27.81 (4.42) |
| Apolipoprotein A, g/L (mean (SD)) | 1.53 (0.26) | 1.49 (0.26) | 1.53 (0.26) | 1.55 (0.26) | 1.55 (0.26) |
| Apolipoprotein B, g/L (mean (SD)) | 1.04 (0.24) | 1.04 (0.24) | 1.04 (0.24) | 1.04 (0.24) | 1.04 (0.24) |
| TG, mmol/L (mean (SD)) | 1.89 (1.05) | 2.01 (1.11) | 1.89 (1.04) | 1.83 (1.02) | 1.82 (1.03) |
| SBP, mmHg (mean (SD)) | 148.96 (15.99) | 147.54 (16.27) | 148.76 (15.96) | 149.42 (15.80) | 150.12 (15.80) |
| HbA1c, % (mean (SD)) | 5.54 (0.68) | 5.61 (0.78) | 5.53 (0.66) | 5.51 (0.65) | 5.51 (0.60) |
| Diabetes, n (%) | 23 052 (10.2) | 7680 (13.6) | 5585 (9.9) | 5138 (9.1) | 4649 (8.2) |
| Depression, n (%) | 70 582 (31.2) | 20 839 (36.8) | 17 456 (30.9) | 16 292 (28.7) | 15 995 (28.4) |
| Parental dementia, n (%) | 26 802 (11.9) | 6468 (11.4) | 6912 (12.2) | 6743 (11.9) | 6679 (11.8) |
| APOE ε4 carriers, n (%) | 53 622 (23.7) | 13 069 (23.1) | 13 464 (23.8) | 13 499 (23.8) | 13 590 (24.1) |

**Abbreviations:** BMI: body mass index; LPA: light physical activity; MET: metabolic equivalent of energy; MPA: moderate physical activity; HbA1c: glycated hemoglobin; SBP: systolic blood pressure; SD: standard deviation; TG: triglyceride; TPA: total physical activity; VPA: vigorous physical activity.

**Table S7. Baseline characteristics in** **early-onset dementia and late-onset dementia analysis in the questionnaire-measured cohort**

| Variables | Early-onset cohort  (n=103 068) | Late-onset cohort  (n=181 769) |
| --- | --- | --- |
| Age, years (mean (SD)) | 51.96 (5.34) | 61.50 (4.89) |
| Male, n (%) | 56 332 (54.7) | 96 263 (53.0) |
| White, n (%) | 95 577 (92.7) | 174 666 (96.1) |
| Townsend deprivation index (mean (SD)) | -1.12 (3.16) | -1.51 (2.98) |
| College/university degree, n (%) | 36 104 (35.0) | 52 316 (28.8) |
| Accelerometer data |  |  |
| Season of accelerometer wearing, n (%) |  |  |
| Spring | 4995 (22.9) | 8207 (22.3) |
| Summer | 5797 (26.6) | 9776 (26.6) |
| Autumn | 6320 (29.0) | 10895 (29.6) |
| Winter | 4675 (21.5) | 7880 (21.4) |
| LPA, MET-min/week (mean (SD)) | 1272.12 (1923.10) | 1196.54 (1659.75) |
| MPA, MET-min/week (mean (SD)) | 962.99 (1815.71) | 1118.61 (1838.36) |
| VPA, MET-min/week (mean (SD)) | 732.44 (1712.64) | 651.14 (1527.90) |
| Lifestyle factors, n (%) |  |  |
| Cigarette smoking |  |  |
| Never | 58 633 (56.9) | 92 352 (50.8) |
| Previous | 32 400 (31.4) | 74 352 (40.9) |
| Current | 12 035 (11.7) | 15 065 (8.3) |
| Healthy alcohol consumption | 80 620 (78.2) | 147 196 (81.0) |
| Ideal diet | 12 866 (12.5) | 29 634 (16.3) |
| Clinical characteristics |  |  |
| BMI, kg/m^2^ (mean (SD)) | 28.81 (5.22) | 28.29 (4.70) |
| Apolipoprotein A, g/L (mean (SD)) | 1.51 (0.26) | 1.54 (0.26) |
| Apolipoprotein B, g/L (mean (SD)) | 1.06 (0.24) | 1.04 (0.24) |
| TG, mmol/L (mean (SD)) | 1.92 (1.13) | 1.88 (1.01) |
| SBP, mmHg (mean (SD)) | 146.60 (14.92) | 149.95 (16.20) |
| HbA1c, % (mean (SD)) | 5.47 (0.69) | 5.57 (0.66) |
| Diabetes, n (%) | 8726 (8.5) | 19 622 (10.8) |
| Depression, n (%) | 37 728 (36.6) | 52 877 (29.1) |
| Parental dementia, n (%) | 9659 (9.4) | 24 043 (13.2) |
| APOE ε4 carriers, n (%) | 24 836 (24.1) | 42 779 (23.5) |
| Incident dementia | 282 (0.27) | 5322 (2.93) |

**Abbreviations:** BMI: body mass index; LPA: light physical activity; MET: metabolic equivalent of energy; MPA: moderate physical activity; HbA1c: glycated hemoglobin; SBP: systolic blood pressure; SD: standard deviation; TG: triglyceride; VPA: vigorous physical activity.

**Table S8. The associations of questionnaire-based physical activity items and incident dementia.**

| PA items |  | Events, n (%) | HR (95% CI) ^a^ | P value |
| --- | --- | --- | --- | --- |
| Leisure time |  |  |  |  |
| Walking | None | 1796(2.81) | Ref |  |
|  | Q1 | 817(2.01) | 0.81(0.75,0.89) | <0.001 |
|  | Q2 | 797(1.88) | 0.73(0.67,0.79) | <0.001 |
|  | Q3 | 994(2.49) | 0.86(0.80,0.93) | <0.001 |
|  | Q4 | 1207(3.11) | 0.94(0.87,1.01) | 0.107 |
| Moderate exercise | None | 3698(2.98) | Ref |  |
|  | Q1 | 566(1.74) | 0.74(0.67,0.80) | <0.001 |
|  | Q2 | 385(1.94) | 0.75(0.67,0.83) | <0.001 |
|  | Q3 | 511(1.84) | 0.74(0.67,0.81) | <0.001 |
|  | Q4 | 455(2.21) | 0.77(0.70,0.86) | <0.001 |
| Strenuous sports | None | 5412(2.62) | Ref |  |
|  | Q1 | 57(1.07) | 0.79(0.61,1.02) | 0.074 |
|  | Q2 | 60(0.95) | 0.64(0.50,0.83) | 0.001 |
|  | Q3 | 45(1.63) | 0.97(0.72,1.30) | 0.843 |
|  | Q4 | 69(1.46) | 0.79(0.62,1.01) | 0.055 |
| Stair climbing | None | 843(3.73) | Ref |  |
|  | ≤10 times/day | 3046(2.36) | 0.87(0.81,0.94) | <0.001 |
|  | >10 times/day | 1714(2.33) | 0.86(0.79,0.94) | <0.001 |
| Housework |  |  |  |  |
| Light DIY | None | 3065(2.73) | Ref |  |
|  | Q1 | 819(2.22) | 0.90(0.83,0.97) | 0.006 |
|  | Q2 | 471(2.07) | 0.86(0.78,0.95) | 0.002 |
|  | Q3 | 425(1.88) | 0.76(0.69,0.84) | <0.001 |
|  | Q4 | 674(2.46) | 0.84(0.77,0.91) | <0.001 |
| Heavy DIY | None | 3396(2.63) | Ref |  |
|  | Q1 | 547(2.31) | 0.81(0.74,0.88) | <0.001 |
|  | Q2 | 484(2.06) | 0.78(0.71,0.86) | <0.001 |
|  | Q3 | 564(2.21) | 0.83(0.76,0.91) | <0.001 |
|  | Q4 | 412(2.33) | 0.75(0.67,0.83) | <0.001 |
| Job |  |  |  |  |
| Mainly walking or standing | Never | 302(0.77) | Ref |  |
|  | Sometimes | 328(0.92) | 1.06(0.90,1.24) | 0.499 |
|  | Usually | 186(1.12) | 1.19(0.99,1.43) | 0.071 |
|  | Always | 244(1.14) | 1.21(1.01,1.44) | 0.034 |
| Involves heavy work | Never | 635(0.87) | Ref |  |
|  | Sometimes | 264(1.07) | 1.09(0.94,1.26) | 0.258 |
|  | Usually | 83(1.06) | 1.03(0.81,1.30) | 0.812 |
|  | Always | 77(1.04) | 1.16(0.91,1.48) | 0.224 |
| Transportation for others |  |  |  |  |
| Car | No | 3787(2.69) | Ref |  |
|  | Yes | 1857(2.17) | 0.96(0.91,1.01) | 0.139 |
| Public | No | 3412(2.17) | Ref |  |
|  | Yes | 2232(3.26) | 1.09(1.03,1.15) | 0.002 |
| Walk | No | 4361(2.59) | Ref |  |
|  | Yes | 1283(2.23) | 0.99(0.93,1.05) | 0.670 |
| Cycle | No | 5396(2.55) | Ref |  |
|  | Yes | 248(1.76) | 0.80(0.71,0.91) | 0.001 |
| Transportation for work |  |  |  |  |
| Car | No | 340(0.91) | Ref |  |
|  | Yes | 597(0.89) | 0.94(0.81,1.08) | 0.355 |
| Public | No | 734(0.88) | Ref |  |
|  | Yes | 203(0.97) | 1.06(0.91,1.25) | 0.454 |
| Walk | No | 848(0.89) | Ref |  |
|  | Yes | 89(0.97) | 1.14(0.91,1.42) | 0.248 |
| Cycle | No | 900(0.92) | Ref |  |
|  | Yes | 37(0.55) | 0.75(0.54,1.04) | 0.089 |

a: Models were adjusted for sex, age at enrollment, ethnicity, Townsend deprivation index, education attainment, lifestyle factors (smoking, healthy drinking, ideal diet), clinical characteristics (BMI, apolipoprotein A, apolipoprotein B, TG, HbA1c, SBP, diabetes, depression, parental dementia, APOE ε4 carriers).

**Abbreviations:** CI: confidence interval; DIY: do-it-yourself; HR: hazard ratio; Ref: reference..

**Table S9. The associations of questionnaire-based physical activity items and incident early-onset dementia and late-onset dementia.**

| PA items |  | Early-onset dementia ^a^ |  |  |  | Late-onset dementia ^a^ |  |  | Ratio of HRs (95% CI) |
| --- | --- | --- | --- | --- | --- | --- | --- | --- | --- |
|  |  | Events, n (%) | HR (95% CI) ^b^ | P value |  | Events, n (%) | HR (95% CI) ^b^ | P value |  |
| Leisure time |  |  |  |  |  |  |  |  |  |
| Walking | None | 109(0.35) | Ref |  |  | 1679(3.41) | Ref |  |  |
|  | Q1 | 40(0.20) | 0.71(0.49,1.03) | 0.070 |  | 942(2.38) | 0.80(0.74,0.87) | <0.001 | 0.89 (0.61, 1.30) |
|  | Q2 | 34(0.19) | 0.69(0.47,1.02) | 0.060 |  | 705(2.40) | 0.77(0.70,0.84) | <0.001 | 0.90 (0.60, 1.34) |
|  | Q3 | 40(0.25) | 0.87(0.60,1.26) | 0.460 |  | 1055(2.90) | 0.87(0.81,0.94) | 0.001 | 1.00 (0.69, 1.45) |
|  | Q4 | 58(0.33) | 1.15(0.83,1.59) | 0.409 |  | 907(3.41) | 0.91(0.84,0.99) | 0.033 | 1.25 (0.90, 1.75) |
| Moderate exercise | None | 189(0.35) | Ref |  |  | 3478(3.41) | Ref |  |  |
|  | Q1 | 27(0.22) | 0.84(0.56,1.27) | 0.407 |  | 534(2.15) | 0.74(0.68,0.81) | <0.001 | 1.13 (0.75, 1.72) |
|  | Q2 | 25(0.21) | 0.84(0.55,1.29) | 0.430 |  | 361(2.30) | 0.74(0.67,0.83) | <0.001 | 1.14 (0.73, 1.76) |
|  | Q3 | 19(0.13) | 0.52(0.32,0.84) | 0.007 |  | 487(2.25) | 0.74(0.67,0.82) | <0.001 | 0.70 (0.43, 1.14) |
|  | Q4 | 20(0.22) | 0.85(0.53,1.36) | 0.493 |  | 432(2.63) | 0.77(0.70,0.85) | <0.001 | 1.10 (0.68, 1.78) |
| Strenuous sports | None | 264(0.29) | Ref |  |  | 5106(3.02) | Ref |  |  |
|  | Q1 | 4(0.13) | 0.65(0.24,1.75) | 0.394 |  | 53(1.59) | 0.83(0.63,1.09) | 0.176 | 0.78 (0.28, 2.19) |
|  | Q2 | 3(0.06) | 0.32(0.10,1.00) | 0.049 |  | 57(1.41) | 0.68(0.52,0.89) | 0.004 | 0.47 (0.14, 1.51) |
|  | Q3 | 4(0.23) | 1.15(0.43,3.12) | 0.777 |  | 49(2.01) | 0.90(0.68,1.19) | 0.467 | 1.28 (0.46, 3.60) |
|  | Q4 | 6(0.22) | 1.05(0.46,2.38) | 0.905 |  | 54(2.08) | 0.79(0.60,1.03) | 0.082 | 1.33 (0.56, 3.15) |
| Stair climbing | None | 42(0.57) | Ref |  |  | 795(4.05) | Ref |  |  |
|  | ≤10 times/day | 166(0.27) | 0.62(0.44,0.88) | 0.007 |  | 2861(2.79) | 0.89(0.82,0.96) | 0.004 | 0.70 (0.49, 1.00) |
|  | >10 times/day | 70(0.21) | 0.57(0.39,0.85) | 0.006 |  | 1626(2.75) | 0.88(0.81,0.96) | 0.004 | 0.65 (0.43, 0.97) |
| Housework |  |  |  |  |  |  |  |  |  |
| Light DIY | None | 170(0.32) | Ref |  |  | 2868(3.23) | Ref |  |  |
|  | Q1 | 31(0.25) | 0.91(0.62,1.33) | 0.623 |  | 777(2.64) | 0.91(0.84,0.98) | 0.017 | 1.00 (0.67, 1.48) |
|  | Q2 | 25(0.20) | 0.80(0.52,1.22) | 0.292 |  | 438(2.43) | 0.85(0.77,0.94) | 0.002 | 0.93 (0.60, 1.44) |
|  | Q3 | 22(0.16) | 0.66(0.42,1.04) | 0.075 |  | 552(2.36) | 0.80(0.73,0.88) | <0.001 | 0.83 (0.52, 1.31) |
|  | Q4 | 27(0.24) | 0.94(0.62,1.43) | 0.784 |  | 501(2.71) | 0.82(0.74,0.90) | <0.001 | 1.15 (0.76, 1.76) |
| Heavy DIY | None | 205(0.33) | Ref |  |  | 3164(3.12) | Ref |  |  |
|  | Q1 | 22(0.22) | 0.78(0.50,1.22) | 0.280 |  | 520(2.64) | 0.81(0.73,0.89) | <0.001 | 0.97 (0.62, 1.53) |
|  | Q2 | 21(0.19) | 0.74(0.47,1.17) | 0.193 |  | 459(2.44) | 0.78(0.71,0.86) | <0.001 | 0.95 (0.59, 1.51) |
|  | Q3 | 13(0.14) | 0.54(0.30,0.94) | 0.031 |  | 546(2.63) | 0.85(0.77,0.93) | 0.001 | 0.63 (0.36, 1.12) |
|  | Q4 | 15(0.16) | 0.56(0.33,0.95) | 0.033 |  | 397(2.66) | 0.75(0.68,0.84) | <0.001 | 0.74 (0.43, 1.28) |
| Job |  |  |  |  |  |  |  |  |  |
| Mainly walking or standing | Never | 38(0.13) | Ref |  |  | 262(1.03) | Ref |  |  |
|  | Sometimes | 38(0.15) | 1.09(0.70,1.71) | 0.704 |  | 290(1.19) | 1.06(0.90,1.26) | 0.478 | 1.03 (0.63, 1.66) |
|  | Usually | 18(0.15) | 1.04(0.59,1.84) | 0.881 |  | 166(1.44) | 1.19(0.98,1.45) | 0.078 | 0.88 (0.48, 1.59) |
|  | Always | 37(0.25) | 1.52(0.95,2.43) | 0.083 |  | 206(1.43) | 1.16(0.97,1.41) | 0.112 | 1.30 (0.78, 2.16) |
| Involves heavy work | Never | 75(0.14) | Ref |  |  | 557(1.13) | Ref |  |  |
|  | Sometimes | 34(0.19) | 1.15(0.76,1.75) | 0.503 |  | 229(1.38) | 1.08(0.92,1.27) | 0.330 | 1.07 (0.68, 1.66) |
|  | Usually | 7(0.13) | 0.69(0.32,1.53) | 0.364 |  | 75(1.44) | 1.07(0.84,1.37) | 0.598 | 0.65 (0.28, 1.48) |
|  | Always | 15(0.27) | 1.41(0.79,2.52) | 0.242 |  | 62(1.35) | 1.12(0.86,1.47) | 0.400 | 1.26 (0.67, 2.38) |
| Transportation for others |  |  |  |  |  |  |  |  |  |
| Car | No | 185(0.31) | Ref |  |  | 3571(3.11) | Ref |  |  |
|  | Yes | 97(0.22) | 0.80(0.62,1.03) | 0.084 |  | 1748(2.61) | 0.97(0.92,1.03) | 0.342 | 0.82 (0.64, 1.07) |
| Public | No | 187(0.23) | Ref |  |  | 3201(2.60) | Ref |  |  |
|  | Yes | 95(0.42) | 1.38(1.06,1.79) | 0.015 |  | 2118(3.63) | 1.08(1.02,1.14) | 0.010 | 1.28 (0.98, 1.67) |
| Walk | No | 207(0.28) | Ref |  |  | 4122(3.03) | Ref |  |  |
|  | Yes | 75(0.26) | 1.01(0.77,1.31) | 0.960 |  | 1197(2.63) | 0.98(0.92,1.05) | 0.542 | 1.03 (0.78, 1.35) |
| Cycle | No | 270(0.28) | Ref |  |  | 5084(2.97) | Ref |  |  |
|  | Yes | 12(0.16) | 0.66(0.37,1.19) | 0.165 |  | 235(2.26) | 0.82(0.71,0.93) | 0.002 | 0.81 (0.45, 1.48) |
| Transportation for work |  |  |  |  |  |  |  |  |  |
| Car | No | 44(0.16) | Ref |  |  | 292(1.21) | Ref |  |  |
|  | Yes | 81(0.17) | 1.14(0.77,1.67) | 0.514 |  | 515(1.14) | 0.92(0.79,1.06) | 0.250 | 1.24 (0.82, 1.87) |
| Public | No | 98(0.16) | Ref |  |  | 634(1.14) | Ref |  |  |
|  | Yes | 27(0.18) | 1.02(0.65,1.58) | 0.945 |  | 173(1.26) | 1.06(0.89,1.26) | 0.527 | 0.96 (0.6, 1.54) |
| Walk | No | 115(0.17) | Ref |  |  | 729(1.16) | Ref |  |  |
|  | Yes | 10(0.15) | 0.82(0.43,1.58) | 0.559 |  | 78(1.25) | 1.19(0.94,1.51) | 0.151 | 0.69 (0.34, 1.39) |
| Cycle | No | 120(0.17) | Ref |  |  | 775(1.18) | Ref |  |  |
|  | Yes | 5(0.09) | 0.58(0.23,1.43) | 0.234 |  | 32(0.86) | 0.80(0.56,1.14) | 0.217 | 0.72 (0.27, 1.91) |

a: Early-onset dementia was defined as dementia diagnosed before 65 years of age, and the analyses were conducted among participants aged younger than 60 years at enrollment. Late-onset dementia was defined as dementia diagnosed at 65 years or older, and the analyses were conducted among individuals aged 65 years or older at the end of follow-up.

b: Models were adjusted for sex, age at enrollment, ethnicity, Townsend deprivation index, education attainment, lifestyle factors (smoking, healthy drinking, ideal diet), clinical characteristics (BMI, apolipoprotein A, apolipoprotein B, TG, HbA1c, SBP, diabetes, depression, parental dementia, APOE ε4 carriers).

**Abbreviations**: CI: confidence interval; DIY: do-it-yourself; HR: hazard ratio; Ref: reference.

**Table S10. Rate advancement periods analyses.**

| PA categories | RAPs, years (95% CI) ^b^ |  |  |  |
| --- | --- | --- | --- | --- |
|  | Accelerometer-measured | Questionnaire-measured |  |  |
|  | Overall | Overall | Early-onset dementia ^a^ | Late-onset dementia ^a^ |
| LPA |  |  |  |  |
| Q1 | Ref | Ref | Ref | Ref |
| Q2 | -1.24(-2.40,-0.08) | 0.03(-0.35,0.41) | -0.53(-6.84,5.79) | -0.03(-0.36,0.30) |
| Q3 | -0.63(-1.79,0.53) | -0.21(-0.62,0.19) | -6.52(-13.7,0.64) | -0.12(-0.47,0.24) |
| Q4 | -0.59(-1.78,0.60) | -0.19(-0.62,0.24) | -4.69(-11.9,2.57) | -0.15(-0.53,0.23) |
| MPA |  |  |  |  |
| Q1 | Ref | Ref | Ref | Ref |
| Q2 | -3.20(-4.46,-1.94) | -1.22(-1.61,-0.82) | -6.62(-13.6,0.41) | -0.89(-1.24,-0.54) |
| Q3 | -3.17(-4.54,-1.79) | -1.10(-1.51,-0.68) | -11.2(-20.0,-2.40) | -0.73(-1.08,-0.38) |
| Q4 | -4.91(-6.67,-3.16) | -1.12(-1.51,-0.73) | -11.2(-19.6,-2.67) | -0.84(-1.18,-0.51) |
| VPA |  |  |  |  |
| Q1 | Ref | Ref | Ref | Ref |
| Q2 | -2.55(-3.74,-1.37) | -1.18(-1.72,-0.64) | -2.86(-11.8,6.12) | -0.64(-1.23,-0.05) |
| Q3 | -3.57(-4.99,-2.15) | -1.19(-1.58,-0.79) | -6.49(-13.8,0.85) | -1.10(-1.43,-0.77) |
| Q4 | -6.01(-7.92,-4.09) | -0.84(-1.21,-0.47) | -2.59(-9.49,4.30) | -0.74(-1.07,-0.42) |
| MVPA |  |  |  |  |
| Q1 | Ref | Ref | Ref | Ref |
| Q2 | -2.77(-3.99,-1.54) | -0.73(-1.12,-0.33) | -5.96(-13.0,1.10) | -0.42(-0.77,-0.07) |
| Q3 | -3.62(-5.06,-2.18) | -1.09(-1.50,-0.69) | -11.2(-20.2,-2.10) | -0.75(-1.10,-0.41) |
| Q4 | -4.68(-6.45,-2.92) | -1.02(-1.43,-0.62) | -7.60(-15.3,0.09) | -0.77(-1.11,-0.42) |
| TPA |  |  |  |  |
| Q1 | Ref | Ref | Ref | Ref |
| Q2 | -1.08(-2.19,0.03) | -0.40(-0.81,0.00) | -3.55(-10.2,3.06) | -0.35(-0.71,0.00) |
| Q3 | -1.30(-2.49,-0.10) | -0.62(-1.02,-0.21) | -9.27(-17.8,-0.78) | -0.53(-0.89,-0.17) |
| Q4 | -2.36(-3.75,-0.96) | -0.64(-1.05,-0.24) | -8.93(-17.1,-0.73) | -0.53(-0.89,-0.17) |

a: Early-onset dementia was defined as dementia diagnosed before 65 years of age, and the analyses were conducted among participants aged younger than 60 years at enrollment. Late-onset dementia was defined as dementia diagnosed at 65 years or older, and the analyses were conducted among individuals aged 65 years or older at the end of follow-up.

b: Analyses were adjusted for sex, age, ethnicity, Townsend deprivation index, education attainment, lifestyle factors (smoking, healthy drinking, ideal diet), clinical characteristics (BMI, apolipoprotein A, apolipoprotein B, TG, HbA1c, SBP, diabetes, depression, parental dementia, APOE ε4 carriers).

**Abbreviations**: CI: confidence interval; LPA: light physical activity; MPA: moderate physical activity; PA: physical activity; RAP: rate advance periods; Ref: reference; TPA: total physical activity; VPA: vigorous physical activity.

**Table S11. The associations between categories of accelerometer-measured physical activity and incident dementia stratified by sex subgroups.**

| PA categories | Female |  |  |  | Male |  |  | P for interaction |
| --- | --- | --- | --- | --- | --- | --- | --- | --- |
|  | Events, n (%) | HR (95% CI) ^a^ | P value |  | Events, n (%) | HR (95% CI) ^a^ | P value |  |
| LPA |  |  |  |  |  |  |  | 0.279 |
| Q1 | 45(1.03) | Ref |  |  | 126(1.66) | Ref |  |  |
| Q2 | 50(0.92) | 0.90(0.60,1.36) | 0.622 |  | 80(1.21) | 0.74(0.56,0.98) | 0.035 |  |
| Q3 | 67(1.08) | 1.06(0.72,1.56) | 0.760 |  | 70(1.22) | 0.79(0.59,1.07) | 0.124 |  |
| Q4 | 79(1.08) | 1.02(0.70,1.50) | 0.902 |  | 57(1.23) | 0.84(0.61,1.15) | 0.284 |  |
| MPA |  |  |  |  |  |  |  | 0.818 |
| Q1 | 115(2.04) | Ref |  |  | 160(2.62) | Ref |  |  |
| Q2 | 51(0.85) | 0.53(0.38,0.74) | <0.001 |  | 76(1.23) | 0.58(0.44,0.76) | <0.001 |  |
| Q3 | 50(0.88) | 0.65(0.46,0.91) | 0.014 |  | 57(0.93) | 0.51(0.37,0.69) | <0.001 |  |
| Q4 | 25(0.42) | 0.37(0.23,0.58) | <0.001 |  | 40(0.65) | 0.45(0.31,0.64) | <0.001 |  |
| VPA |  |  |  |  |  |  |  | 0.325 |
| Q1 | 127(1.84) | Ref |  |  | 138(2.74) | Ref |  |  |
| Q2 | 68(1.09) | 0.72(0.53,0.97) | 0.032 |  | 83(1.45) | 0.58(0.44,0.76) | <0.001 |  |
| Q3 | 34(0.63) | 0.51(0.35,0.75) | 0.001 |  | 68(1.06) | 0.53(0.39,0.72) | <0.001 |  |
| Q4 | 12(0.25) | 0.26(0.14,0.48) | <0.001 |  | 44(0.59) | 0.37(0.26,0.52) | <0.001 |  |
| MVPA |  |  |  |  |  |  |  | 0.525 |
| Q1 | 117(1.96) | Ref |  |  | 159(2.65) | Ref |  |  |
| Q2 | 61(1.03) | 0.65(0.48,0.90) | 0.008 |  | 74(1.23) | 0.57(0.43,0.75) | <0.001 |  |
| Q3 | 39(0.68) | 0.52(0.36,0.76) | 0.001 |  | 59(0.96) | 0.52(0.38,0.71) | <0.001 |  |
| Q4 | 24(0.43) | 0.39(0.25,0.62) | <0.001 |  | 41(0.64) | 0.46(0.32,0.66) | <0.001 |  |
| TPA |  |  |  |  |  |  |  | 0.399 |
| Q1 | 67(1.40) | Ref |  |  | 137(1.91) | Ref |  |  |
| Q2 | 59(1.04) | 0.78(0.54,1.11) | 0.167 |  | 92(1.46) | 0.84(0.64,1.09) | 0.194 |  |
| Q3 | 63(1.03) | 0.86(0.60,1.22) | 0.391 |  | 65(1.11) | 0.73(0.54,0.98) | 0.037 |  |
| Q4 | 52(0.78) | 0.70(0.48,1.03) | 0.071 |  | 39(0.74) | 0.58(0.40,0.83) | 0.003 |  |

a: Models were adjusted for age, ethnicity, Townsend deprivation index, education attainment, lifestyle factors (smoking, healthy drinking, ideal diet), clinical characteristics (BMI, apolipoprotein A, apolipoprotein B, TG, HbA1c, SBP, diabetes, depression, parental dementia, APOE ε4 carriers).

**Abbreviations**: CI: confidence interval; HR: hazard ratio; LPA: light physical activity; MPA: moderate physical activity; PA: physical activity; Ref: reference; TPA: total physical activity; VPA: vigorous physical activity.

**Table S12. The associations between categories of accelerometer-measured physical activity and incident dementia stratified by age subgroups.**

| PA categories | Age < 65 y |  |  |  | Age ≥ 65 y |  |  | P for interaction |
| --- | --- | --- | --- | --- | --- | --- | --- | --- |
|  | Events, n (%) | HR (95% CI) ^a^ | P value |  | Events, n (%) | HR (95% CI) ^a^ | P value |  |
| LPA |  |  |  |  |  |  |  | 0.061 |
| Q1 | 23(0.41) | Ref |  |  | 148(2.34) | Ref |  |  |
| Q2 | 11(0.20) | 0.52(0.25,1.07) | 0.074 |  | 119(1.84) | 0.82(0.64,1.04) | 0.107 |  |
| Q3 | 14(0.25) | 0.70(0.36,1.38) | 0.300 |  | 123(1.97) | 0.91(0.71,1.16) | 0.453 |  |
| Q4 | 11(0.19) | 0.55(0.26,1.15) | 0.115 |  | 125(1.99) | 0.93(0.73,1.20) | 0.597 |  |
| MPA |  |  |  |  |  |  |  | <0.001 |
| Q1 | 30(0.79) | Ref |  |  | 245(3.08) | Ref |  |  |
| Q2 | 13(0.24) | 0.36(0.18,0.69) | 0.002 |  | 114(1.67) | 0.58(0.47,0.73) | <0.001 |  |
| Q3 | 12(0.20) | 0.31(0.15,0.62) | 0.001 |  | 95(1.65) | 0.60(0.47,0.77) | <0.001 |  |
| Q4 | 4(0.05) | 0.09(0.03,0.26) | <0.001 |  | 61(1.28) | 0.49(0.37,0.66) | <0.001 |  |
| VPA |  |  |  |  |  |  |  | 0.083 |
| Q1 | 24(0.57) | Ref |  |  | 241(3.11) | Ref |  |  |
| Q2 | 14(0.28) | 0.52(0.27,1.01) | 0.055 |  | 137(1.94) | 0.64(0.52,0.79) | <0.001 |  |
| Q3 | 17(0.28) | 0.56(0.29,1.06) | 0.075 |  | 85(1.47) | 0.51(0.40,0.66) | <0.001 |  |
| Q4 | 4(0.05) | 0.12(0.04,0.35) | <0.001 |  | 52(1.10) | 0.39(0.29,0.54) | <0.001 |  |
| MVPA |  |  |  |  |  |  |  | <0.001 |
| Q1 | 30(0.78) | Ref |  |  | 246(3.02) | Ref |  |  |
| Q2 | 13(0.25) | 0.37(0.19,0.71) | 0.003 |  | 122(1.80) | 0.64(0.51,0.79) | <0.001 |  |
| Q3 | 13(0.21) | 0.32(0.16,0.64) | 0.001 |  | 85(1.46) | 0.54(0.42,0.70) | <0.001 |  |
| Q4 | 3(0.04) | 0.07(0.02,0.22) | <0.001 |  | 62(1.36) | 0.53(0.40,0.71) | <0.001 |  |
| TPA |  |  |  |  |  |  |  | 0.001 |
| Q1 | 25(0.51) | Ref |  |  | 179(2.53) | Ref |  |  |
| Q2 | 17(0.32) | 0.71(0.38,1.33) | 0.291 |  | 134(2.01) | 0.82(0.65,1.03) | 0.089 |  |
| Q3 | 11(0.19) | 0.44(0.21,0.92) | 0.028 |  | 117(1.88) | 0.83(0.65,1.06) | 0.128 |  |
| Q4 | 6(0.09) | 0.23(0.09,0.58) | 0.002 |  | 85(1.58) | 0.71(0.54,0.93) | 0.013 |  |

a: Models were adjusted for sex, ethnicity, Townsend deprivation index, education attainment, lifestyle factors (smoking, healthy drinking, ideal diet), clinical characteristics (BMI, apolipoprotein A, apolipoprotein B, TG, HbA1c, SBP, diabetes, depression, parental dementia, APOE ε4 carriers).

**Abbreviations**: CI: confidence interval; HR: hazard ratio; LPA: light physical activity; MPA: moderate physical activity; PA: physical activity; Ref: reference; TPA: total physical activity; VPA: vigorous physical activity.

**Table S13. The associations between categories of accelerometer-measured physical activity and incident dementia stratified by APOE ε4 carriers subgroups.**

| PA categories | APOE ε4 carriers |  |  |  | No APOE ε4 |  |  | P for interaction |
| --- | --- | --- | --- | --- | --- | --- | --- | --- |
|  | Events, n (%) | HR (95% CI) ^a^ | P value |  | Events, n (%) | HR (95% CI) ^a^ | P value |  |
| LPA |  |  |  |  |  |  |  | 0.063 |
| Q1 | 64(2.37) | Ref |  |  | 103(1.15) | Ref |  |  |
| Q2 | 57(2.07) | 0.89(0.62,1.28) | 0.538 |  | 70(0.77) | 0.72(0.53,0.97) | 0.032 |  |
| Q3 | 63(2.28) | 1.03(0.72,1.47) | 0.863 |  | 68(0.76) | 0.75(0.55,1.03) | 0.078 |  |
| Q4 | 67(2.36) | 1.06(0.74,1.52) | 0.749 |  | 67(0.76) | 0.79(0.57,1.09) | 0.144 |  |
| MPA |  |  |  |  |  |  |  | 0.045 |
| Q1 | 111(4.16) | Ref |  |  | 157(1.78) | Ref |  |  |
| Q2 | 50(1.81) | 0.56(0.40,0.79) | 0.001 |  | 74(0.81) | 0.56(0.42,0.74) | <0.001 |  |
| Q3 | 60(2.18) | 0.79(0.57,1.09) | 0.149 |  | 45(0.51) | 0.41(0.29,0.58) | <0.001 |  |
| Q4 | 30(1.05) | 0.47(0.31,0.72) | 0.001 |  | 32(0.35) | 0.35(0.23,0.52) | <0.001 |  |
| VPA |  |  |  |  |  |  |  | 0.765 |
| Q1 | 118(4.30) | Ref |  |  | 140(1.57) | Ref |  |  |
| Q2 | 64(2.33) | 0.61(0.45,0.83) | 0.002 |  | 83(0.92) | 0.66(0.50,0.86) | 0.003 |  |
| Q3 | 44(1.66) | 0.51(0.36,0.73) | <0.001 |  | 56(0.63) | 0.54(0.39,0.75) | <0.001 |  |
| Q4 | 25(0.86) | 0.34(0.21,0.53) | <0.001 |  | 29(0.32) | 0.34(0.22,0.51) | <0.001 |  |
| MVPA |  |  |  |  |  |  |  | 0.150 |
| Q1 | 111(4.13) | Ref |  |  | 158(1.76) | Ref |  |  |
| Q2 | 61(2.22) | 0.69(0.50,0.95) | 0.021 |  | 71(0.80) | 0.55(0.42,0.74) | <0.001 |  |
| Q3 | 48(1.75) | 0.64(0.45,0.91) | 0.013 |  | 47(0.52) | 0.43(0.31,0.60) | <0.001 |  |
| Q4 | 31(1.08) | 0.50(0.33,0.77) | 0.001 |  | 32(0.36) | 0.37(0.25,0.55) | <0.001 |  |
| TPA |  |  |  |  |  |  |  | 0.080 |
| Q1 | 77(2.90) | Ref |  |  | 123(1.36) | Ref |  |  |
| Q2 | 70(2.53) | 0.94(0.68,1.31) | 0.718 |  | 76(0.85) | 0.70(0.53,0.94) | 0.018 |  |
| Q3 | 57(2.09) | 0.90(0.63,1.28) | 0.555 |  | 67(0.74) | 0.69(0.51,0.94) | 0.018 |  |
| Q4 | 47(1.62) | 0.78(0.53,1.14) | 0.202 |  | 42(0.48) | 0.52(0.36,0.76) | 0.001 |  |

a: Models were adjusted for sex, age, ethnicity, Townsend deprivation index, education attainment, lifestyle factors (smoking, healthy drinking, ideal diet), clinical characteristics (BMI, apolipoprotein A, apolipoprotein B, TG, HbA1c, SBP, diabetes, depression, parental dementia).

**Abbreviations**: CI: confidence interval; HR: hazard ratio; LPA: light physical activity; MPA: moderate physical activity; PA: physical activity; Ref: reference; TPA: total physical activity; VPA: vigorous physical activity.

**Table S14. The associations between categories of accelerometer-measured physical activity and incident dementia stratified by years of hypertension subgroups.**

| PA categories | Prevalent hypertension ≥ 10 y |  |  |  | Prevalent hypertension < 10 y |  |  | P for interaction |
| --- | --- | --- | --- | --- | --- | --- | --- | --- |
|  | Events, n (%) | HR (95% CI) ^a^ | P value |  | Events, n (%) | HR (95% CI) ^a^ | P value |  |
| LPA |  |  |  |  |  |  |  | 0.952 |
| Q1 | 75(1.88) | Ref |  |  | 96(1.21) | Ref |  |  |
| Q2 | 49(1.32) | 0.71(0.50,1.02) | 0.068 |  | 81(0.97) | 0.83(0.62,1.12) | 0.230 |  |
| Q3 | 48(1.40) | 0.77(0.53,1.12) | 0.167 |  | 89(1.05) | 0.98(0.73,1.31) | 0.886 |  |
| Q4 | 52(1.57) | 0.85(0.59,1.24) | 0.406 |  | 84(0.97) | 0.93(0.69,1.27) | 0.655 |  |
| MPA |  |  |  |  |  |  |  | 0.130 |
| Q1 | 117(2.57) | Ref |  |  | 158(2.20) | Ref |  |  |
| Q2 | 44(1.15) | 0.51(0.36,0.72) | <0.001 |  | 83(0.99) | 0.59(0.45,0.77) | <0.001 |  |
| Q3 | 40(1.23) | 0.63(0.43,0.92) | 0.015 |  | 67(0.78) | 0.52(0.39,0.70) | <0.001 |  |
| Q4 | 23(0.81) | 0.47(0.29,0.75) | 0.001 |  | 42(0.45) | 0.39(0.27,0.56) | <0.001 |  |
| VPA |  |  |  |  |  |  |  | 0.105 |
| Q1 | 109(2.46) | Ref |  |  | 156(2.08) | Ref |  |  |
| Q2 | 55(1.42) | 0.61(0.44,0.85) | 0.003 |  | 96(1.18) | 0.64(0.49,0.83) | 0.001 |  |
| Q3 | 40(1.21) | 0.62(0.42,0.90) | 0.011 |  | 62(0.73) | 0.47(0.35,0.64) | <0.001 |  |
| Q4 | 20(0.71) | 0.39(0.24,0.64) | <0.001 |  | 36(0.39) | 0.32(0.22,0.46) | <0.001 |  |
| MVPA |  |  |  |  |  |  |  | 0.118 |
| Q1 | 117(2.52) | Ref |  |  | 159(2.17) | Ref |  |  |
| Q2 | 48(1.28) | 0.57(0.40,0.80) | 0.001 |  | 87(1.07) | 0.63(0.48,0.82) | 0.001 |  |
| Q3 | 36(1.09) | 0.57(0.39,0.84) | 0.004 |  | 62(0.72) | 0.49(0.36,0.66) | <0.001 |  |
| Q4 | 23(0.83) | 0.51(0.31,0.81) | 0.005 |  | 42(0.45) | 0.40(0.28,0.57) | <0.001 |  |
| TPA |  |  |  |  |  |  |  | 0.242 |
| Q1 | 84(1.94) | Ref |  |  | 120(1.57) | Ref |  |  |
| Q2 | 62(1.65) | 0.89(0.64,1.24) | 0.489 |  | 89(1.08) | 0.76(0.58,1.01) | 0.057 |  |
| Q3 | 44(1.30) | 0.77(0.53,1.11) | 0.162 |  | 84(0.98) | 0.78(0.59,1.04) | 0.097 |  |
| Q4 | 34(1.14) | 0.72(0.47,1.09) | 0.124 |  | 57(0.64) | 0.60(0.43,0.84) | 0.003 |  |

a: Models were adjusted for sex, age, ethnicity, Townsend deprivation index, education attainment, lifestyle factors (smoking, healthy drinking, ideal diet), clinical characteristics (BMI, apolipoprotein A, apolipoprotein B, TG, HbA1c, SBP, diabetes, depression, parental dementia, APOE ε4 carriers).

**Abbreviations**: CI: confidence interval; HR: hazard ratio; LPA: light physical activity; MPA: moderate physical activity; PA: physical activity; Ref: reference; TPA: total physical activity; VPA: vigorous physical activity.

**Table S15. The associations between categories of questionnaire-measured physical activity and incident dementia stratified by sex subgroups.**

| PA categories | Female |  |  |  | Male |  |  | P for interaction |
| --- | --- | --- | --- | --- | --- | --- | --- | --- |
|  | Events, n (%) | HR (95% CI) ^a^ | P value |  | Events, n (%) | HR (95% CI) ^a^ | P value |  |
| LPA |  |  |  |  |  |  |  | 0.069 |
| Q1 | 587(2.05) | Ref |  |  | 886(2.55) | Ref |  |  |
| Q2 | 741(2.32) | 0.99(0.89,1.11) | 0.876 |  | 1029(2.98) | 1.01(0.93,1.11) | 0.749 |  |
| Q3 | 591(2.43) | 1.06(0.94,1.19) | 0.353 |  | 743(2.65) | 0.90(0.82,0.99) | 0.037 |  |
| Q4 | 425(2.23) | 0.99(0.88,1.13) | 0.923 |  | 645(2.60) | 0.94(0.85,1.05) | 0.262 |  |
| MPA |  |  |  |  |  |  |  | 0.163 |
| Q1 | 611(2.36) | Ref |  |  | 909(2.95) | Ref |  |  |
| Q2 | 597(1.88) | 0.81(0.73,0.91) | <0.001 |  | 867(2.38) | 0.79(0.72,0.87) | <0.001 |  |
| Q3 | 501(2.26) | 0.84(0.75,0.95) | 0.005 |  | 680(2.68) | 0.80(0.72,0.89) | <0.001 |  |
| Q4 | 635(2.62) | 0.84(0.75,0.95) | 0.003 |  | 847(2.86) | 0.79(0.72,0.87) | <0.001 |  |
| VPA |  |  |  |  |  |  |  | 0.633 |
| Q1 | 1275(2.59) | Ref |  |  | 1621(3.30) | Ref |  |  |
| Q2 | 167(1.74) | 0.76(0.65,0.89) | 0.001 |  | 298(2.39) | 0.83(0.74,0.94) | 0.004 |  |
| Q3 | 436(1.88) | 0.80(0.72,0.90) | <0.001 |  | 603(2.30) | 0.81(0.74,0.89) | <0.001 |  |
| Q4 | 466(2.11) | 0.88(0.79,0.99) | 0.026 |  | 781(2.28) | 0.84(0.77,0.92) | <0.001 |  |
| MVPA |  |  |  |  |  |  |  | 0.138 |
| Q1 | 604(2.26) | Ref |  |  | 929(3.03) | Ref |  |  |
| Q2 | 613(2.16) | 0.90(0.80,1.01) | 0.066 |  | 817(2.74) | 0.86(0.78,0.95) | 0.002 |  |
| Q3 | 581(2.18) | 0.85(0.76,0.95) | 0.006 |  | 738(2.44) | 0.79(0.72,0.88) | <0.001 |  |
| Q4 | 546(2.44) | 0.87(0.77,0.98) | 0.021 |  | 819(2.60) | 0.80(0.73,0.88) | <0.001 |  |
| TPA |  |  |  |  |  |  |  | 0.032 |
| Q1 | 552(2.08) | Ref |  |  | 881(2.94) | Ref |  |  |
| Q2 | 613(2.27) | 1.03(0.92,1.15) | 0.645 |  | 777(2.64) | 0.87(0.79,0.95) | 0.004 |  |
| Q3 | 601(2.28) | 0.98(0.87,1.10) | 0.749 |  | 782(2.57) | 0.84(0.76,0.92) | <0.001 |  |
| Q4 | 578(2.40) | 0.97(0.86,1.09) | 0.618 |  | 863(2.68) | 0.84(0.76,0.92) | <0.001 |  |

a: Models were adjusted for age, ethnicity, Townsend deprivation index, education attainment, lifestyle factors (smoking, healthy drinking, ideal diet), clinical characteristics (BMI, apolipoprotein A, apolipoprotein B, TG, HbA1c, SBP, diabetes, depression, parental dementia, APOE ε4 carriers).

**Abbreviations**: CI: confidence interval; HR: hazard ratio; LPA: light physical activity; MPA: moderate physical activity; PA: physical activity; Ref: reference; TPA: total physical activity; VPA: vigorous physical activity.

**Table S16. The associations between categories of questionnaire-measured physical activity and incident dementia stratified by APOE ε4 carriers subgroups.**

| PA categories | APOE ε4 carriers |  |  |  | No APOE ε4 |  |  | P for interaction |
| --- | --- | --- | --- | --- | --- | --- | --- | --- |
|  | Events, n (%) | HR (95% CI) ^a^ | P value |  | Events, n (%) | HR (95% CI) ^a^ | P value |  |
| LPA |  |  |  |  |  |  |  | 0.002 |
| Q1 | 574(3.92) | Ref |  |  | 853(1.80) | Ref |  |  |
| Q2 | 753(4.80) | 1.05(0.94,1.17) | 0.382 |  | 976(1.97) | 0.99(0.90,1.09) | 0.833 |  |
| Q3 | 599(4.81) | 1.04(0.93,1.17) | 0.465 |  | 699(1.80) | 0.91(0.82,1.01) | 0.067 |  |
| Q4 | 491(4.71) | 1.08(0.95,1.22) | 0.236 |  | 557(1.71) | 0.90(0.81,1.00) | 0.059 |  |
| MPA |  |  |  |  |  |  |  | 0.018 |
| Q1 | 587(4.46) | Ref |  |  | 880(2.09) | Ref |  |  |
| Q2 | 635(3.98) | 0.87(0.77,0.97) | 0.013 |  | 794(1.56) | 0.77(0.70,0.84) | <0.001 |  |
| Q3 | 541(4.79) | 0.90(0.80,1.02) | 0.090 |  | 615(1.74) | 0.77(0.69,0.85) | <0.001 |  |
| Q4 | 654(5.12) | 0.86(0.77,0.96) | 0.010 |  | 796(1.99) | 0.79(0.72,0.87) | <0.001 |  |
| VPA |  |  |  |  |  |  |  | 0.448 |
| Q1 | 1207(5.34) | Ref |  |  | 1608(2.19) | Ref |  |  |
| Q2 | 205(3.89) | 0.85(0.73,0.99) | 0.035 |  | 250(1.53) | 0.78(0.69,0.90) | 0.003 |  |
| Q3 | 450(3.80) | 0.80(0.72,0.90) | <0.001 |  | 565(1.55) | 0.81(0.73,0.89) | <0.001 |  |
| Q4 | 555(4.11) | 0.87(0.79,0.97) | 0.008 |  | 662(1.58) | 0.85(0.77,0.93) | <0.001 |  |
| MVPA |  |  |  |  |  |  |  | 0.080 |
| Q1 | 601(4.58) | Ref |  |  | 879(2.05) | Ref |  |  |
| Q2 | 625(4.56) | 0.93(0.83,1.05) | 0.233 |  | 773(1.79) | 0.85(0.77,0.94) | 0.001 |  |
| Q3 | 581(4.29) | 0.84(0.75,0.95) | 0.004 |  | 708(1.68) | 0.81(0.73,0.90) | <0.001 |  |
| Q4 | 610(4.75) | 0.87(0.77,0.97) | 0.015 |  | 725(1.81) | 0.81(0.73,0.90) | <0.001 |  |
| TPA |  |  |  |  |  |  |  | 0.050 |
| Q1 | 564(4.36) | Ref |  |  | 829(1.96) | Ref |  |  |
| Q2 | 597(4.47) | 0.96(0.85,1.08) | 0.475 |  | 752(1.79) | 0.91(0.82,1.00) | 0.053 |  |
| Q3 | 609(4.55) | 0.93(0.83,1.05) | 0.251 |  | 739(1.75) | 0.86(0.78,0.95) | 0.004 |  |
| Q4 | 647(4.79) | 0.93(0.83,1.04) | 0.205 |  | 765(1.83) | 0.87(0.78,0.96) | 0.005 |  |

a: Models were adjusted for sex, age, ethnicity, Townsend deprivation index, education attainment, lifestyle factors (smoking, healthy drinking, ideal diet), clinical characteristics (BMI, apolipoprotein A, apolipoprotein B, TG, HbA1c, SBP, diabetes, depression, parental dementia).

**Abbreviations**: CI: confidence interval; HR: hazard ratio; LPA: light physical activity; MPA: moderate physical activity; PA: physical activity; Ref: reference; TPA: total physical activity; VPA: vigorous physical activity.

**Table S17. The associations between categories of questionnaire-measured physical activity and incident dementia stratified by years of hypertension subgroups.**

| PA categories | Prevalent hypertension ≥ 10 y |  |  |  | Prevalent hypertension < 10 y |  |  | P for interaction |
| --- | --- | --- | --- | --- | --- | --- | --- | --- |
|  | Events, n (%) | HR (95% CI) ^a^ | P value |  | Events, n (%) | HR (95% CI) ^a^ | P value |  |
| LPA |  |  |  |  |  |  |  | 0.705 |
| Q1 | 428(3.60) | Ref |  |  | 1033(2.03) | Ref |  |  |
| Q2 | 420(3.52) | 0.88(0.77,1.01) | 0.075 |  | 1339(2.48) | 1.06(0.97,1.15) | 0.189 |  |
| Q3 | 312(3.41) | 0.85(0.73,0.99) | 0.032 |  | 1012(2.37) | 1.01(0.92,1.10) | 0.871 |  |
| Q4 | 260(3.63) | 0.94(0.81,1.10) | 0.472 |  | 804(2.21) | 0.98(0.90,1.08) | 0.744 |  |
| MPA |  |  |  |  |  |  |  | 0.259 |
| Q1 | 417(3.97) | Ref |  |  | 1093(2.40) | Ref |  |  |
| Q2 | 378(3.20) | 0.81(0.71,0.94) | 0.004 |  | 1075(1.93) | 0.79(0.73,0.86) | <0.001 |  |
| Q3 | 286(3.38) | 0.78(0.67,0.90) | 0.001 |  | 887(2.29) | 0.83(0.76,0.91) | <0.001 |  |
| Q4 | 339(3.62) | 0.77(0.67,0.89) | 0.001 |  | 1133(2.57) | 0.83(0.76,0.90) | <0.001 |  |
| VPA |  |  |  |  |  |  |  | 0.965 |
| Q1 | 759(3.98) | Ref |  |  | 2108(2.69) | Ref |  |  |
| Q2 | 132(3.35) | 0.94(0.78,1.13) | 0.49 |  | 332(1.84) | 0.77(0.68,0.86) | 0.001 |  |
| Q3 | 249(2.98) | 0.83(0.71,0.95) | 0.009 |  | 786(1.93) | 0.80(0.74,0.87) | <0.001 |  |
| Q4 | 280(3.18) | 0.86(0.74,0.98) | 0.028 |  | 962(2.04) | 0.87(0.80,0.94) | <0.001 |  |
| MVPA |  |  |  |  |  |  |  | 0.592 |
| Q1 | 426(3.87) | Ref |  |  | 1098(2.40) | Ref |  |  |
| Q2 | 357(3.42) | 0.87(0.75,1.00) | 0.047 |  | 1060(2.24) | 0.87(0.80,0.95) | 0.002 |  |
| Q3 | 344(3.49) | 0.86(0.74,0.99) | 0.037 |  | 963(2.07) | 0.80(0.73,0.87) | <0.001 |  |
| Q4 | 293(3.31) | 0.78(0.67,0.91) | 0.001 |  | 1067(2.39) | 0.85(0.78,0.92) | <0.001 |  |
| TPA |  |  |  |  |  |  |  | 0.828 |
| Q1 | 414(3.78) | Ref |  |  | 1009(2.24) | Ref |  |  |
| Q2 | 326(3.24) | 0.85(0.73,0.98) | 0.026 |  | 1054(2.29) | 0.96(0.88,1.04) | 0.318 |  |
| Q3 | 358(3.64) | 0.90(0.78,1.04) | 0.152 |  | 1011(2.18) | 0.89(0.81,0.97) | 0.007 |  |
| Q4 | 322(3.47) | 0.84(0.73,0.98) | 0.026 |  | 1114(2.39) | 0.91(0.83,0.99) | 0.034 |  |

a: Models were adjusted for sex, age, ethnicity, Townsend deprivation index, education attainment, lifestyle factors (smoking, healthy drinking, ideal diet), clinical characteristics (BMI, apolipoprotein A, apolipoprotein B, TG, HbA1c, SBP, diabetes, depression, parental dementia, APOE ε4 carriers).

**Abbreviations**: CI: confidence interval; HR: hazard ratio; LPA: light physical activity; MPA: moderate physical activity; PA: physical activity; Ref: reference; TPA: total physical activity; VPA: vigorous physical activity.

**Table S18. The associations between categories of questionnaire-measured physical activity and incident dementia stratified by baseline systolic blood pressure subgroups.**

| PA categories | SBP ≥ 160 mmHg |  |  |  | SBP < 160 mmHg |  |  | P for interaction |
| --- | --- | --- | --- | --- | --- | --- | --- | --- |
|  | Events, n (%) | HR (95% CI) ^a^ | P value |  | Events, n (%) | HR (95% CI) ^a^ | P value |  |
| LPA |  |  |  |  |  |  |  | 0.614 |
| Q1 | 333(2.67) | Ref |  |  | 1140(2.24) | Ref |  |  |
| Q2 | 462(3.26) | 1.07(0.93,1.23) | 0.342 |  | 1308(2.50) | 0.99(0.91,1.07) | 0.715 |  |
| Q3 | 365(3.19) | 1.03(0.88,1.19) | 0.719 |  | 969(2.37) | 0.94(0.86,1.03) | 0.168 |  |
| Q4 | 270(2.74) | 0.91(0.77,1.07) | 0.235 |  | 800(2.35) | 0.99(0.90,1.08) | 0.83 |  |
| MPA |  |  |  |  |  |  |  | 0.995 |
| Q1 | 373(3.36) | Ref |  |  | 1147(2.52) | Ref |  |  |
| Q2 | 325(2.35) | 0.81(0.71,0.94) | 0.004 |  | 1139(2.10) | 0.84(0.77,0.91) | <0.001 |  |
| Q3 | 324(3.10) | 0.78(0.67,0.90) | 0.001 |  | 857(2.31) | 0.82(0.75,0.90) | <0.001 |  |
| Q4 | 408(3.25) | 0.77(0.67,0.89) | 0.001 |  | 1074(2.60) | 0.83(0.76,0.91) | <0.001 |  |
| VPA |  |  |  |  |  |  |  | 0.766 |
| Q1 | 714(3.49) | Ref |  |  | 2182(2.80) | Ref |  |  |
| Q2 | 110(2.37) | 0.94(0.78,1.13) | 0.49 |  | 355(2.03) | 0.82(0.73,0.91) | 0.001 |  |
| Q3 | 254(2.39) | 0.83(0.71,0.95) | 0.009 |  | 785(2.03) | 0.83(0.76,0.90) | <0.001 |  |
| Q4 | 352(2.88) | 0.86(0.74,0.98) | 0.028 |  | 895(2.03) | 0.85(0.78,0.92) | <0.001 |  |
| MVPA |  |  |  |  |  |  |  | 0.643 |
| Q1 | 371(3.33) | Ref |  |  | 1162(2.52) | Ref |  |  |
| Q2 | 341(2.81) | 0.87(0.75,1.00) | 0.047 |  | 1089(2.37) | 0.91(0.83,0.98) | 0.019 |  |
| Q3 | 335(2.71) | 0.86(0.74,0.99) | 0.037 |  | 984(2.21) | 0.85(0.78,0.92) | <0.001 |  |
| Q4 | 383(3.11) | 0.78(0.67,0.91) | 0.001 |  | 982(2.36) | 0.84(0.77,0.92) | <0.001 |  |
| TPA |  |  |  |  |  |  |  | 0.824 |
| Q1 | 330(3.04) | Ref |  |  | 1103(2.41) | Ref |  |  |
| Q2 | 340(2.88) | 0.85(0.73,0.98) | 0.026 |  | 1050(2.35) | 0.94(0.87,1.03) | 0.171 |  |
| Q3 | 365(2.95) | 0.90(0.78,1.04) | 0.152 |  | 1018(2.30) | 0.90(0.83,0.99) | 0.022 |  |
| Q4 | 395(3.06) | 0.84(0.73,0.98) | 0.026 |  | 1046(2.41) | 0.90(0.83,0.98) | 0.020 |  |

a: Models were adjusted for sex, age, ethnicity, Townsend deprivation index, education attainment, lifestyle factors (smoking, healthy drinking, ideal diet), clinical characteristics (BMI, apolipoprotein A, apolipoprotein B, TG, HbA1c, diabetes, depression, parental dementia, APOE ε4 carriers).

**Abbreviations**: CI: confidence interval; HR: hazard ratio; LPA: light physical activity; MPA: moderate physical activity; PA: physical activity; Ref: reference; SBP: systolic blood pressure; TPA: total physical activity; VPA: vigorous physical activity.

**Table S19. Associations between categories of accelerometer-measured physical activity or questionnaire-measured physical activity and incident dementia using competing risk model.**

| PA categories | Accelerometer-measured |  |  | Questionnaire-measured |  |
| --- | --- | --- | --- | --- | --- |
|  | HR (95% CI) ^a^ | P value |  | HR (95% CI) ^a^ | P value |
| LPA |  |  |  |  |  |
| Q1 | Ref |  |  | Ref |  |
| Q2 | 0.79(0.63,1.00) | 0.051 |  | 1.02(0.96,1.10) | 0.487 |
| Q3 | 0.91(0.72,1.14) | 0.403 |  | 0.99(0.92,1.06) | 0.717 |
| Q4 | 0.90(0.71,1.14) | 0.397 |  | 1.00(0.92,1.08) | 0.955 |
| P for trend |  | 0.589 |  |  | 0.697 |
| MPA |  |  |  |  |  |
| Q1 | Ref |  |  | Ref |  |
| Q2 | 0.57(0.46,0.71) | <0.001 |  | 0.82(0.76,0.88) | <0.001 |
| Q3 | 0.58(0.46,0.74) | <0.001 |  | 0.84(0.78,0.91) | <0.001 |
| Q4 | 0.42(0.32,0.57) | <0.001 |  | 0.84(0.78,0.90) | <0.001 |
| P for trend |  | <0.001 |  |  | <0.001 |
| VPA |  |  |  |  |  |
| Q1 | Ref |  |  | Ref |  |
| Q2 | 0.65(0.53,0.79) | <0.001 |  | 0.83(0.75,0.91) | <0.001 |
| Q3 | 0.54(0.43,0.69) | <0.001 |  | 0.83(0.77,0.89) | <0.001 |
| Q4 | 0.35(0.26,0.48) | <0.001 |  | 0.89(0.83,0.95) | <0.001 |
| P for trend |  | <0.001 |  |  | <0.001 |
| MVPA |  |  |  |  |  |
| Q1 | Ref |  |  | Ref |  |
| Q2 | 0.62(0.50,0.77) | <0.001 |  | 0.90(0.83,0.96) | 0.003 |
| Q3 | 0.54(0.42,0.69) | <0.001 |  | 0.85(0.79,0.91) | <0.001 |
| Q4 | 0.44(0.33,0.60) | <0.001 |  | 0.86(0.80,0.93) | <0.001 |
| P for trend |  | <0.001 |  |  | <0.001 |
| TPA |  |  |  |  |  |
| Q1 | Ref |  |  | Ref |  |
| Q2 | 0.83(0.67,1.04) | 0.102 |  | 0.96(0.89,1.03) | 0.239 |
| Q3 | 0.80(0.64,1.01) | 0.060 |  | 0.92(0.86,1.00) | 0.037 |
| Q4 | 0.65(0.50,0.85) | 0.002 |  | 0.92(0.86,1.00) | 0.037 |
| P for trend |  | 0.002 |  |  | 0.026 |

a: Models were conducted using competitive risk models and adjusted for sex, age, ethnicity, Townsend deprivation index, education attainment, lifestyle factors (smoking, healthy drinking, ideal diet), clinical characteristics (BMI, apolipoprotein A, apolipoprotein B, TG, HbA1c, SBP, diabetes, depression, parental dementia, APOE ε4 carriers).

**Abbreviations**: CI: confidence interval; HR: hazard ratio; LPA: light physical activity; MPA: moderate physical activity; PA: physical activity; Ref: reference; TPA: total physical activity; VPA: vigorous physical activity.

**Table S20. Associations between categories of accelerometer-measured physical activity or questionnaire-measured physical activity and incident dementia among participants with complete covariates.**

| PA categories | Accelerometer-measured |  |  |  | Questionnaire-measured |  |  |
| --- | --- | --- | --- | --- | --- | --- | --- |
|  | Events, n (%) | HR (95% CI) ^a^ | P value |  | Events, n (%) | HR (95% CI) ^a^ | P value |
| LPA |  |  |  |  |  |  |  |
| Q1 | 132(1.45) | Ref |  |  | 1099(2.26) | Ref |  |
| Q2 | 99(1.06) | 0.75(0.58,0.98) | 0.034 |  | 1321(2.61) | 1.00(0.93,1.09) | 0.909 |
| Q3 | 95(1.02) | 0.77(0.59,1.01) | 0.057 |  | 1006(2.51) | 0.97(0.89,1.06) | 0.559 |
| Q4 | 99(1.08) | 0.82(0.62,1.07) | 0.148 |  | 806(2.40) | 0.99(0.90,1.08) | 0.778 |
| P for trend |  |  | 0.158 |  |  |  | 0.604 |
| MPA |  |  |  |  |  |  |  |
| Q1 | 212(2.37) | Ref |  |  | 1120(2.61) | Ref |  |
| Q2 | 88(0.94) | 0.49(0.38,0.63) | <0.001 |  | 1100(2.11) | 0.81(0.75,0.88) | <0.001 |
| Q3 | 78(0.85) | 0.51(0.39,0.67) | <0.001 |  | 896(2.46) | 0.83(0.76,0.91) | <0.001 |
| Q4 | 47(0.50) | 0.37(0.27,0.52) | <0.001 |  | 1116(2.70) | 0.82(0.75,0.89) | <0.001 |
| P for trend |  |  | <0.001 |  |  |  | <0.001 |
| VPA |  |  |  |  |  |  |  |
| Q1 | 199(2.18) | Ref |  |  | 2122(2.85) | Ref |  |
| Q2 | 115(1.24) | 0.64(0.50,0.80) | <0.001 |  | 370(2.17) | 0.86(0.77,0.96) | 0.008 |
| Q3 | 72(0.79) | 0.49(0.37,0.65) | <0.001 |  | 779(2.06) | 0.81(0.75,0.88) | <0.001 |
| Q4 | 39(0.41) | 0.31(0.21,0.44) | <0.001 |  | 961(2.21) | 0.89(0.82,0.96) | 0.003 |
| P for trend |  |  | <0.001 |  |  |  | <0.001 |
| MVPA |  |  |  |  |  |  |  |
| Q1 | 212(2.33) | Ref |  |  | 1119(2.58) | Ref |  |
| Q2 | 92(1.00) | 0.53(0.41,0.68) | <0.001 |  | 1064(2.40) | 0.89(0.82,0.97) | 0.009 |
| Q3 | 74(0.80) | 0.50(0.38,0.65) | <0.001 |  | 1012(2.32) | 0.85(0.78,0.93) | <0.001 |
| Q4 | 47(0.50) | 0.39(0.28,0.55) | <0.001 |  | 1037(2.50) | 0.85(0.78,0.93) | <0.001 |
| P for trend |  |  | <0.001 |  |  |  | <0.001 |
| TPA |  |  |  |  |  |  |  |
| Q1 | 155(1.70) | Ref |  |  | 1061(2.47) | Ref |  |
| Q2 | 118(1.27) | 0.81(0.64,1.03) | 0.091 |  | 1029(2.38) | 0.93(0.85,1.01) | 0.086 |
| Q3 | 85(0.92) | 0.66(0.50,0.87) | 0.003 |  | 1051(2.42) | 0.91(0.84,0.99) | 0.037 |
| Q4 | 67(0.73) | 0.59(0.44,0.80) | 0.001 |  | 1091(2.52) | 0.91(0.83,0.99) | 0.026 |
| P for trend |  |  | <0.001 |  |  |  | 0.029 |

a: Models were adjusted for sex, age, ethnicity, Townsend deprivation index, education attainment, lifestyle factors (smoking, healthy drinking, ideal diet), clinical characteristics (BMI, apolipoprotein A, apolipoprotein B, TG, HbA1c, SBP, diabetes, depression, parental dementia, APOE ε4 carriers).

**Abbreviations**: CI: confidence interval; HR: hazard ratio; LPA: light physical activity; MPA: moderate physical activity; PA: physical activity; Ref: reference; TPA: total physical activity; VPA: vigorous physical activity.

**Table S21. Associations between categories of accelerometer-measured physical activity or questionnaire-measured physical activity and incident dementia after exclusion of participants within 2 years of dementia or death onset.**

| PA categories | Accelerometer-measured |  |  |  | Questionnaire-measured |  |  |
| --- | --- | --- | --- | --- | --- | --- | --- |
|  | Events, n (%) | HR (95% CI) ^a^ | P value |  | Events, n (%) | HR (95% CI) ^a^ | P value |
| LPA |  |  |  |  |  |  |  |
| Q1 | 165(1.40) | Ref |  |  | 1450(2.30) | Ref |  |
| Q2 | 118(0.99) | 0.73(0.58,0.93) | 0.010 |  | 1743(2.64) | 1.01(0.94,1.08) | 0.888 |
| Q3 | 124(1.05) | 0.82(0.65,1.04) | 0.100 |  | 1315(2.53) | 0.96(0.89,1.04) | 0.318 |
| Q4 | 128(1.08) | 0.86(0.67,1.09) | 0.211 |  | 1057(2.42) | 0.97(0.89,1.05) | 0.426 |
| P for trend |  |  | 0.303 |  |  |  | 0.250 |
| MPA |  |  |  |  |  |  |  |
| Q1 | 260(2.26) | Ref |  |  | 1492(2.66) | Ref |  |
| Q2 | 119(0.99) | 0.55(0.44,0.68) | <0.001 |  | 1443(2.13) | 0.80(0.75,0.86) | <0.001 |
| Q3 | 98(0.83) | 0.54(0.42,0.68) | <0.001 |  | 1169(2.47) | 0.82(0.76,0.89) | <0.001 |
| Q4 | 58(0.48) | 0.38(0.28,0.51) | <0.001 |  | 1461(2.73) | 0.82(0.76,0.88) | <0.001 |
| P for trend |  |  | <0.001 |  |  |  | <0.001 |
| VPA |  |  |  |  |  |  |  |
| Q1 | 249(2.12) | Ref |  |  | 2852(2.92) | Ref |  |
| Q2 | 142(1.19) | 0.62(0.51,0.77) | <0.001 |  | 462(2.10) | 0.81(0.74,0.90) | <0.001 |
| Q3 | 92(0.78) | 0.50(0.39,0.63) | <0.001 |  | 1017(2.07) | 0.80(0.74,0.86) | <0.001 |
| Q4 | 52(0.43) | 0.33(0.24,0.45) | <0.001 |  | 1234(2.20) | 0.86(0.80,0.92) | <0.001 |
| P for trend |  |  | <0.001 |  |  |  | <0.001 |
| MVPA |  |  |  |  |  |  |  |
| Q1 | 261(2.22) | Ref |  |  | 1506(2.65) | Ref |  |
| Q2 | 127(1.07) | 0.60(0.48,0.74) | <0.001 |  | 1411(2.44) | 0.88(0.82,0.94) | <0.001 |
| Q3 | 89(0.75) | 0.49(0.38,0.63) | <0.001 |  | 1297(2.29) | 0.82(0.76,0.88) | <0.001 |
| Q4 | 58(0.48) | 0.40(0.29,0.53) | <0.001 |  | 1351(2.52) | 0.83(0.77,0.90) | <0.001 |
| P for trend |  |  | <0.001 |  |  |  | <0.001 |
| TPA |  |  |  |  |  |  |  |
| Q1 | 198(1.68) | Ref |  |  | 1408(2.51) | Ref |  |
| Q2 | 135(1.14) | 0.74(0.59,0.92) | 0.008 |  | 1369(2.44) | 0.93(0.86,1.00) | 0.059 |
| Q3 | 118(0.99) | 0.73(0.58,0.92) | 0.008 |  | 1363(2.42) | 0.89(0.83,0.96) | 0.004 |
| Q4 | 84(0.71) | 0.59(0.45,0.77) | <0.001 |  | 1425(2.54) | 0.89(0.83,0.96) | 0.003 |
| P for trend |  |  | <0.001 |  |  |  | 0.002 |

a: Models were adjusted for sex, age, ethnicity, Townsend deprivation index, education attainment, lifestyle factors (smoking, healthy drinking, ideal diet), clinical characteristics (BMI, apolipoprotein A, apolipoprotein B, TG, HbA1c, SBP, diabetes, depression, parental dementia, APOE ε4 carriers).

**Abbreviations**: CI: confidence interval; HR: hazard ratio; LPA: light physical activity; MPA: moderate physical activity; PA: physical activity; Ref: reference; TPA: total physical activity; VPA: vigorous physical activity.

**Table S22. Associations between categories of accelerometer-measured physical activity and incident dementia after changing the threshold of MPA to more than 100 mg.**

| PA categories | Events, n (%) | HR (95% CI) ^a^ | P value |
| --- | --- | --- | --- |
| LPA |  |  |  |
| ≤4756.75 | 166(1.38) | Ref |  |
| 4756.75-5621.62 | 114(0.96) | 0.70(0.55,0.88) | 0.003 |
| 5621.62-6486.48 | 139(1.16) | 0.89(0.70,1.11) | 0.302 |
| >6486.48 | 155(1.29) | 0.96(0.76,1.21) | 0.729 |
| P for trend |  |  | 0.838 |
| MPA ^b^ |  |  |  |
| ≤1769.82 | 266(2.22) | Ref |  |
| 1769.82-2478.41 | 132(1.10) | 0.62(0.50,0.76) | <0.001 |
| 2478.41-3306.24 | 105(0.87) | 0.56(0.45,0.71) | <0.001 |
| >3306.24 | 71(0.60) | 0.47(0.36,0.62) | <0.001 |
| P for trend |  |  | <0.001 |
| MVPA |  |  |  |
| ≤1854.72 | 271(2.30) | Ref |  |
| 1854.72-2645.91 | 132(1.09) | 0.58(0.47,0.71) | <0.001 |
| 2645.91-3588.48 | 102(0.84) | 0.53(0.42,0.67) | <0.001 |
| >3588.48 | 69(0.58) | 0.45(0.34,0.60) | <0.001 |
| P for trend |  |  | <0.001 |
| TPA |  |  |  |
| ≤6939.32 | 203(1.70) | Ref |  |
| 6939.32-8396.64 | 153(1.28) | 0.83(0.67,1.02) | 0.078 |
| 8396.64-9911.66 | 128(1.07) | 0.79(0.63,0.99) | 0.038 |
| >9911.66 | 90(0.75) | 0.63(0.48,0.82) | <0.001 |
| P for trend |  |  | <0.001 |

a: Models were adjusted for sex, age, ethnicity, Townsend deprivation index, education attainment, lifestyle factors (smoking, healthy drinking, ideal diet), clinical characteristics (BMI, apolipoprotein A, apolipoprotein B, TG, HbA1c, SBP, diabetes, depression, parental dementia, APOE ε4 carriers).

b: The threshold of MPA change from 125 mg to 100 mg.

**Abbreviations**: CI: confidence interval; HR: hazard ratio; LPA: light physical activity; MPA: moderate physical activity; PA: physical activity; Ref: reference; TPA: total physical activity.

**Table S23. Associations between accelerometer-measured or questionnaire-measured LPA, MPA, VPA and incident dementia after including LPA, MPA, VPA to mutually adjust each other.**

| PA categories | Accelerometer-measured |  |  | Questionnaire-measured |  |
| --- | --- | --- | --- | --- | --- |
|  | HR (95% CI) ^a^ | P value |  | HR (95% CI) ^a^ | P value |
| LPA |  |  |  |  |  |
| Q1 | Ref |  |  | Ref |  |
| Q2 | 0.97(0.77,1.23) | 0.814 |  | 1.04(0.97,1.12) | 0.267 |
| Q3 | 1.22(0.96,1.55) | 0.111 |  | 1.00(0.93,1.08) | 0.946 |
| Q4 | 1.38(1.06,1.79) | 0.015 |  | 1.02(0.93,1.11) | 0.707 |
| P for trend |  | 0.016 |  |  | 0.471 |
| MPA |  |  |  |  |  |
| Q1 | Ref |  |  | Ref |  |
| Q2 | 0.62(0.49,0.78) | <0.001 |  | 0.83(0.77,0.90) | <0.001 |
| Q3 | 0.68(0.52,0.90) | 0.006 |  | 0.86(0.79,0.93) | <0.001 |
| Q4 | 0.55(0.39,0.78) | 0.001 |  | 0.86(0.79,0.94) | <0.001 |
| P for trend |  | 0.001 |  |  | 0.003 |
| VPA |  |  |  |  |  |
| Q1 | Ref |  |  | Ref |  |
| Q2 | 0.70(0.57,0.88) | 0.002 |  | 0.84(0.76,0.92) | 0.001 |
| Q3 | 0.64(0.49,0.84) | 0.001 |  | 0.84(0.78,0.91) | <0.001 |
| Q4 | 0.43(0.30,0.61) | <0.001 |  | 0.89(0.83,0.96) | 0.003 |
| P for trend |  | <0.001 |  |  | <0.001 |

a: Models were adjusted for sex, age, ethnicity, Townsend deprivation index, education attainment, lifestyle factors (smoking, healthy drinking, ideal diet), clinical characteristics (BMI, apolipoprotein A, apolipoprotein B, TG, HbA1c, SBP, diabetes, depression, parental dementia, APOE ε4 carriers), physical activity (LPA, MPA, VPA).

**Abbreviations**: CI: confidence interval; HR: hazard ratio; LPA: light physical activity; MPA: moderate physical activity; PA: physical activity; Ref: reference; TPA: total physical activity; VPA: vigorous physical activity.

**Supplementary Figures:**

**Figure S1**. Nonlinear associations between accelerometer-measured physical activity or questionnaire-measured physical activity and incident dementia.

Models were adjusted for sex, age, ethnicity, Townsend deprivation index, education attainment, lifestyle factors (smoking, healthy drinking, ideal diet), clinical characteristics (BMI, apolipoprotein A, apolipoprotein B, TG, HbA1c, SBP, diabetes, depression, parental dementia, APOE ε4 carriers). The minimum value of each exposure was used as a reference group and the knots were at the 25th, 50th, and 75th centiles.

Abbreviations: LPA: light physical activity; MPA: moderate physical activity; PA: physical activity.

**Figure S2.** Nonlinear associations between questionnaire-measured physical activity and incident early-onset dementia and late-onset dementia.

Models were adjusted for sex, age, ethnicity, Townsend deprivation index, education attainment, lifestyle factors (smoking, healthy drinking, ideal diet), clinical characteristics (BMI, apolipoprotein A, apolipoprotein B, TG, HbA1c, SBP, diabetes, depression, parental dementia, APOE ε4 carriers). The minimum value of each exposure was used as a reference group and the knots were at the 25th, 50th, and 75th centiles.

Abbreviations: LPA: light physical activity; MPA: moderate physical activity; PA: physical activity.

**References**

[1]	Fan M, Lyu J, He P. [Chinese guidelines for data processing and analysis concerning the International Physical Activity Questionnaire]. Zhonghua Liu Xing Bing Xue Za Zhi. 2014. 35(8): 961-4.

[2]	Brenner H, Gefeller O, Greenland S. Risk and rate advancement periods as measures of exposure impact on the occurrence of chronic diseases. Epidemiology. 1993. 4(3): 229-36.

[3]	Pfahlberg AB, Gefeller O, Brenner H. Point and Interval Estimation of Risk and Rate Advancement Periods in Epidemiologic Studies Using the SAS ® Software. 2011.

[4]	Huxley RR, Woodward M. Cigarette smoking as a risk factor for coronary heart disease in women compared with men: a systematic review and meta-analysis of prospective cohort studies. Lancet. 2011. 378(9799): 1297-305.

[5]	Li R, Li R, Xie J, et al. Associations of socioeconomic status and healthy lifestyle with incident early-onset and late-onset dementia: a prospective cohort study. Lancet Healthy Longev. 2023. 4(12): e693-e702.
